# Cap-dependent translation initiation monitored in living cells

**DOI:** 10.1038/s41467-022-34052-8

**Published:** 2022-11-02

**Authors:** Valentina Gandin, Brian P. English, Melanie Freeman, Louis-Philippe Leroux, Stephan Preibisch, Deepika Walpita, Maritza Jaramillo, Robert H. Singer

**Affiliations:** 1 Janelia Research Campus, Howard Hughes Medical Institute, Ashburn, VA USA; 2 Institut National de la Recherche Scientifique (INRS)-Centre Armand-Frappier Santé Biotechnologie (CAFSB), Laval, QC Canada

**Keywords:** Translation, Cell biology

## Abstract

mRNA translation is tightly regulated to preserve cellular homeostasis. Despite extensive biochemical, genetic, and structural studies, a detailed understanding of mRNA translation regulation is lacking. Imaging methodologies able to resolve the binding dynamics of translation factors at single-cell and single-mRNA resolution were necessary to fully elucidate regulation of this paramount process. Here live-cell spectroscopy and single-particle tracking were combined to interrogate the binding dynamics of endogenous initiation factors to the 5’cap. The diffusion of initiation factors (IFs) changed markedly upon their association with mRNA. Quantifying their diffusion characteristics revealed the sequence of IFs assembly and disassembly in cell lines and the clustering of translation in neurons. This approach revealed translation regulation at high spatial and temporal resolution that can be applied to the formation of any endogenous complex that results in a measurable shift in diffusion.

## Abstract (web summary)

mRNA translation is tightly regulated to preserve cellular homeostasis. Here live-cell spectroscopy and single-particle tracking are combined to interrogate the binding dynamics of endogenous initiation factors to mRNAs 5’cap, revealing the sequence of translation initiation factors assembly and disassembly; and the clustering of translation in neurons.

## Introduction

Despite three decades of extensive biochemical and genetic studies, our understanding of translation regulation remains incomplete. The ability to detect the dynamic behavior of individual translation factors in live cells at a sufficient spatiotemporal resolution to understand how mRNA translation is dynamically regulated is essential to determine the cell’s response to extracellular and intracellular signals to preserve homeostasis.

Initiation of cap-dependent translation is a rate-limiting step in translation. The best characterized regulatory step is the assembly of eIF4F complexes on the 5’cap structure of the mRNA^1^. The eIF4F complex is composed of the 5’cap-binding protein eIF4Es, eIF4Gs, and the helicase eIF4As. It is not known whether in the living cells the eIF4F complexes bind “en masse” to the 5’cap or whether eIF4E binds the mRNA first to recruit the other initiation factors. It is well established, that the eIF4F assembly is regulated by the mechanistic target of rapamycin complex 1 (mTORC1)^2,3^. mTORC1 phosphorylates a family of inhibitory proteins called 4E-BPs^2^. When phosphorylated, 4E-BPs do not bind eIF4E. Since 4E-BPs and eIF4Gs share the same binding site on eIF4Es, at initiation eIF4G binds and stabilizes eIF4E on the 5’cap^3^. Unlike phosphorylation, the mechanism underlying the de-phosphorylation of 4E-BPs remains largely unknown. Protein phosphatase PPM1G dephosphorylate 4E-BP1 during conditions that impaired mTOR activity. It is not known whether the cycle of phosphorylation–dephosphorylation of 4E-BPs occurs at each re-initiation event. Our platform applied the techniques of fluorescence correlation and cross-correlation spectroscopy (FCS, FCCS) and single-particle tracking (SPT) to interrogate the binding of initiation factors to the 5’cap, with single-cell and single-molecule resolution. To achieve this, we fused tags suitable for live imaging to endogenous translation initiation factors eIF4E, eIF4G, and 4E-BP1 homozygously without affecting their function.

FCS provides ensemble-averaged diffusion characteristics of thousands of molecules (from pM to nM) in a few seconds, localized to a diffraction-limited volume within the cell^4^. Moreover, dual-fluorescence cross-correlation spectroscopy (FCCS) can measure the diffusion of two molecules labeled with different fluorophores. Because diffusion characteristics are recorded simultaneously, temporal cross-correlation can reveal the degree of binding between two molecules^5^. SPT gives a detailed overview of molecular diffusion with single-molecule resolution (from fM to pM). Here we combine these two orthogonal approaches to challenge the robustness of our findings. These powerful methodologies are capable of resolving early molecular events of translation initiation on the mRNA in living cells.

By labeling individual endogenous molecules of the initiation factors eIF4E and eIF4G, we were able to detect changes in their diffusion by FCS and SPT when they bind to the mRNA as well as when they are assembled on the 5’cap. Using the same methodology, we investigated the diffusional behavior of the translation repressor 4E-BP1 and its effect on eIF4E, monitoring in real-time the dissociation of the eIF4F complexes from the mRNA to inhibit translation. We were able to elucidate translation regulation in the cytoplasm of dividing cells as well as in neuronal processes, with single mRNA resolution. Our platform can be used to accurately elucidate the underpinnings of mRNA translation in the native environment.

## Results

### Dynamic detection of eIF4E bound to the 5’cap of mRNAs

FCS reveals the timescale of fluctuations as fluorescent molecules diffuse through the focal volume in crowded conditions. Therefore, we reasoned that FCS had the potential to resolve differential diffusion of initiation factors, mRNA-bound vs. -unbound, despite their high concentration in living cells. The focal volume is in the order of femtoliters with detection limits ranging from 100 pM to 100 nM. Since the diffusion of thousands of molecules is averaged in tens of seconds, autocorrelations determine the diffusion of molecules with excellent statistical confidence in a short time, presumably without locally perturbing mRNA translation.

The Halo-tag was fused to the non-conserved N-terminal region of eIF4E that is dispensable for eIF4G binding^6^. Fusion of the Halo-tag to eIF4E did not result in any degradation products that could lead to artefactual changes in eIF4E diffusion in live imaging (Supplementary Fig. 1a). Since eIF4E is an essential gene^7^, cells stop proliferating when it is suppressed. Halo-eIF4E was made insensitive to eIF4E shRNA by synonymous changes in the coding sequence and was expressed in NIH3T3 fibroblasts in which the endogenous counterpart was silenced by shRNA (Supplementary Fig. 1b). Exogenous Halo-eIF4E was able to bind the 5’cap and rescue cell proliferation in NIH3T3 cells (Supplementary Fig 1c, d). These data demonstrated that the Halo tag did not perturb the eIF4E function. We then determined whether FCS could resolve _JF-646_Halo-eIF4E binding to the 5’cap in living NIH3T3. Prolonged slow fluctuations in the fluorescent signal, corresponding to slow-moving molecules throughout the focal volume, were observed in the cytoplasm, but not in the nucleus where translation did not occur (Supplementary Fig 2a, c). Autocorrelations confirmed that two eIF4E components existed in the cytoplasm of translating cells. The fast component had a diffusion time of ~1 ms (*D* = 17 μm^2^/s), which is expected for free molecules, whereas the slow component had a diffusion time of ~300 ms (*D* = 0.06 μm^2^/s).

**Fig. 1:**
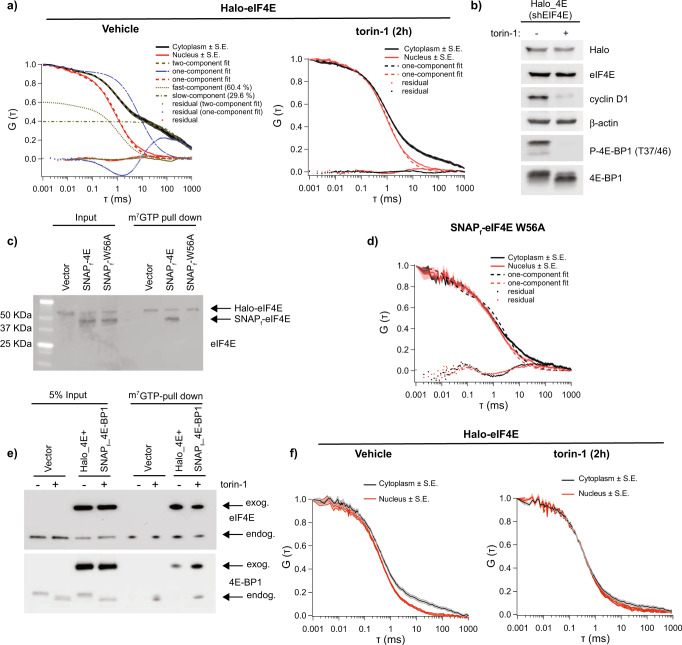
Binding of exogenous Halo-eIF4E to the 5’cap is detected by FCS. **a** NIH3T3 cells that express Halo-eIF4E, in which the endogenous counterpart was silenced by shRNA, were treated for 2 h with vehicle (DMSO) or 250 nM torin-1. Averaged autocorrelation curves show temporal diffusion of Halo-eIF4E molecules (ms = milliseconds) in the cytoplasm (black) and in the nucleus (red) in the indicated conditions (*N* = 10 ± SEM). Halo-eIF4E diffusion is slower in translating cells as compared to cells treated with torin-1. Cytoplasmic autocorrelation best fit with two components (fast – dark yellow dotted curve: *τ*_fast_ = 1.14 ± 0.03 ms, and slow – dash-dotted curve: *τ*_slow_ = 372.69 ± 10.8 ms). Percentages of slow (*D*_slow_ = 0.05 μm^2^/s) and fast (*D*_fast_ = 14.78 μm^2^/s) moving molecules are indicated. While one-component fits nuclear Halo-eIF4E well (see red curves), a one-component fit (see the blue dashed curve, with dotted fit residual) cannot adequately describe the cytoplasmic Halo-eIF4E autocorrelation curve. **b** Total cell lysates from the cells described in (**a**) were analyzed by western blotting with the indicated antibodies. 4E-BPs phosphorylation and CyclinD1 expression were significantly reduced after 2 h torin-1. **c**, **d** SNAP_f_-eIF4E and SNAP_f_-eIF4E W56A (SNAP_f_-W56A) were expressed in the cells described above. Total cell lysates were subjected to a cap-pull-down assay. Levels of Halo-eIF4E, SNAP_f_-eIF4E, and SNAP_f_-eIF4E W56A were detected by western blotting in input (5%) and cap-bound fractions (**c**) using eIF4E antibody (eIF4E). Averaged autocorrelation curves show temporal diffusion of SNAP_f_-eIF4E W56A in the cytoplasm (red) and in the nucleus (black) (*N* = 10 ± SEM). Only one fast component was detected in both cellular compartments (**d**). **e**, **f** NIH3T3 cells that express both Halo-eIF4E and SNAP_f_-4E-BP1 were treated with vehicle (DMSO) or 250 nM torin-1 for 2 h. Total cell lysates were subjected to cap-pull down assay and analyzed by western blotting in the indicated fractions Overexpression of SNAP_f_-4E-BP1 is sufficient to increase its binding to eIF4E on the 5’cap (**e**). Averaged autocorrelation curves show temporal diffusion of Halo-eIF4E in the indicated conditions (*N* = 10 ± SEM). SNAP_f_-4E-BP1 expression is sufficient to displace most of the Halo-eIF4E bound to the 5’cap (**f**).

The diffusion parameters confirmed that the mRNA-bound molecules diffused on average more than two orders of magnitude slower than the unbound counterpart. Most important, this magnitude in diffusion was expected when free molecules bound to a slow-moving mRNA^8^. Only one fast-component was detected in the cytoplasm or nucleus (Fig. 1a right, Supplementary Fig. 2b, d) after a two-hour treatment with the mTOR inhibitor torin-1, which promotes dephosphorylation of 4E-BP1 whereupon it binds to eIF4E and inhibits eIF4E binding to the 5’ cap, thus preventing initiation. This inhibition was confirmed by a decrease in the expression of Cyclin D1, a canonical eIF4E-sensitive mRNA^9^ (Fig. 1b).

In order to verify that the slower component of eIF4E diffusion in the autocorrelation was due to its binding to the 5’cap of the mRNA, the eIF4E^W56A^ mutant was monitored in living cells. Changing tryptophan 56 to alanine perturbs the electrostatic interactions to the 7-methyl-guanine of the 5’cap preventing eIF4E from binding to the mRNA^10,11^. The shRNA-resistant Halo-eIF4E and either the SNAP_f_-eIF4E or the SNAP_f_-eIF4E^W56A^ were co-expressed in NIH3T3 in which their endogenous counterpart had been silenced by shRNA. As expected, the wild-type version of SNAP-tagged eIF4E bound the 5’cap, while the Trp56Ala mutation abolished the binding to the 5’cap (Fig. 1c). Autocorrelations of cytoplasmic and nuclear SNAP_f_-eIF4E^W56A^ revealed only one-fast component in translating cells (Fig. 1d), consistent with the conclusion that the slow diffusion of eIF4E in translating cells was due to its binding to the mRNA.

**Fig. 2:**
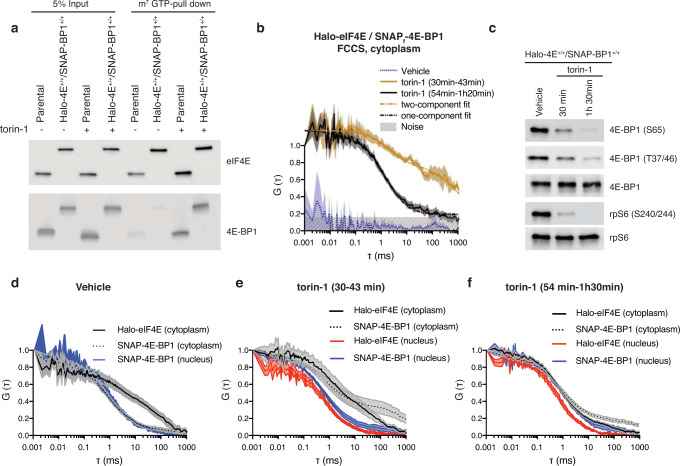
eIF4E is released from the 5’cap upon binding to 4E-BP1. **a** Differentiated parental and mESC in which Halo and SNAP_f_ tags were inserted into the EIF4E and 4EBP1 locus, respectively (Halo-4E^+/+^/SNAP-BP1^+/+^), were treated with vehicle (DMSO) or 250 nM torin-1 for 1 h and 30 min. Total cell lysates (Input) were subjected to cap-pull-down assay and analyzed by western blotting using the indicated antibodies. The Halo and SNAP_f_ tags do not affect eIF4E:4E-BP1 binding upon mTOR inhibition. **b** mESC double knock-in described in (**a**) were treated with vehicle (DMSO) or 250 nM torin-1. Simultaneous diffusion of _JF585_Halo-eIF4E and _JF646_SNAP_f_-4E-BP1 was analyzed by dual color cross-correlation spectroscopy in the indicated conditions. Cross-correlation was detected, in the cytoplasm, 30 to 1 h 20 min upon mTOR inhibition and with differential diffusion speed. The vehicle showed no correlation over time (gray) (*N* = 10 ± SEM). **c** mESC double knock-in described in (**a**) was treated with vehicle (DMSO) or 250 nM torin-1 for 30 or 90 min. Total cell lysates were analyzed by western blotting using the indicated antibodies. 4E-BP1 and rpS6 were used as loading controls. **d**–**f** Individual diffusion of _JF585_Halo-eIF4E and _JF646_SNAP_f_-4E-BP1 was analyzed by FCS in control cells (vehicle) (**d**) or in torin-1 treated cells (**e**, **f**). Averaged autocorrelation curves show two Halo-eIF4E components (fast and slow) and one-fast SNAP_f_-4E-BP1 component in the cytoplasm of translating cells. The nuclear diffusion of SNAP_f_-4E-BP1 is depicted in blue (**d**). Upon 30–43 min torin-1 treatment, SNAP_f_-4E-BP1 diffusion slows down in the cytoplasm with Halo-eIF4E still moving slower than its nuclear counterpart (**e**). After 54–90 min torin-1 treatment, both Halo-eIF4E and SNAP_f_-4E-BP1 autocorrelations show overall fast diffusion (**f**) (*N* = 10 ± SEM).

The eIF4F complex formation is inhibited in the cytoplasm by 4E-BP’s binding to eIF4E upon mTOR inhibition^2^. We sought to determine whether the dissociation of eIF4E from the 5’cap was triggered by 4E-BP1 binding. SNAP_f_ tag was fused to the N-terminus of 4E-BP1, in order to preserve the TOR signaling (TOS) motif at the C-terminus. 4E-BP1/eIF4E stoichiometry determines the sensitivity to active-site mTOR inhibitors, which are more efficient with higher 4E-BPs levels^12^. To assess whether the SNAP_f_ tag perturbed 4E-BP1 function, an shRNA-resistant SNAP_f_-4E-BP1 was expressed in NIH3T3 in which the endogenous counterpart was depleted by shRNA (Supplementary. Fig. 2a). 4E-BP1 overexpression attenuated cell proliferation compared to the vector control, with or without the endogenous knockdown (Supplementary Fig. 3b). NIH3T3 cells that expressed vector control or SNAP_f_−4E-BP1 were treated with vehicle control or torin-1 for 16 h (Supplementary Fig. 3c). The cytostatic effect of torin-1 was more pronounced in NIH3T3 that expressed SNAP_f_-4E-BP1, due to its enhancement of cap-binding and consequent competition with eIF4E. These data demonstrated that the SNAP_f_ tag did not perturb the 4E-BP1 function. In vitro cap-binding assays confirmed that overexpression of SNAP_f_−4E-BP1 was sufficient to increase its binding to eIF4E, with a more pronounced interaction upon mTOR inhibition (Fig. 1e). In living cells, FCS revealed that SNAP_f_−4E-BP1 overexpression correlated with _JF646_Halo-eIF4E dissociation from the 5’cap, that was further increased upon mTOR inhibition (Fig. 1f).

To avoid experimental variation in eIF4E:4E-BP1 stoichiometry that affect translation regulation, Halo and SNAP_f_ tags were inserted into the endogenous *Eif4e* and *Eif4epb1* loci, respectively, using Crispr/Cas9 in mouse Embryonic Stem Cells (mESC) (Fig. 2a). Since cap-dependent translation is more pronounced throughout mESC differentiation^13^, parental and double knock-in (DKI) Halo-eIF4E^+/+^/SNAP_f_-4E-BP1^+/+^ mESC were differentiated into fibroblasts (not shown). mTOR inhibition promoted eIF4E binding to 4E-BP1 in both parental and DKI with no major differences. We then examined the diffusional behavior of _JF646_SNAP_f_-4E-BP1 and _JF585_Halo-eIF4E by fluorescence cross-correlation spectroscopy (FCCS). We simultaneously recorded the diffusion of both fluorescent tags through the focal volume for thousands of molecules. FCCS detected the simultaneous diffusion of the two fluorescent tags (Fig. 2b). The photon counts as a function of time for the two fluorescent channels were correlated with each other, the amplitude of which reflected their simultaneous occupancy in the same complex^5,14^. The cross-correlation analysis demonstrated almost no correlation in translating cells, indicating that the tagged molecules were not interacting (Fig. 2b, vehicle). After 30 min of mTOR inhibition, partial 4E-BP1 dephosphorylation was observed (Fig. 2c) with _JF646_SNAP_f_-4E-BP1 and _JF585_Halo-eIF4E molecules interacting together with the mRNA as demonstrated by the slow component in the cross-correlations (Fig. 2b). After 1 h, mTOR inhibition led to a more pronounced 4E-BP1 dephosphorylation (Fig. 2c) with the _JF585_Halo-eIF4E: _JF646_SNAP_f_-4E-BP1 slow component of the complexes (representing co-binding to the mRNA) almost eliminated (Fig. 2b). These data indicate that 4E-BP1 initially bound eIF4E at the 5’cap to compete with eIF4G and this event triggered subsequent eIF4E dissociation from the mRNA. Autocorrelations for single _JF646_SNAP_f_-4E-BP1 and _JF585_Halo-eIF4E signals demonstrated that _JF646_SNAP_f_-4E-BP1 diffusion significantly slowed down in the cytoplasm after 30-min with simultaneous changes in the diffusion of _JF585_Halo-eIF4E molecules compared to control cells (Fig. 2d, e). At 30 min, a slow eIF4E component is still detected within the cells indicative of eIF4E:4E-BP1 complexes binding the mRNA. As expected, mTOR inhibition did not perturb _JF646_SNAP_f_-4E-BP1 and _JF585_Halo-eIF4E diffusion in the nucleus, indicating that the binding events occurred on the mRNA to inhibit translation initiation. After 1 h, FCS revealed that both _JF646_SNAP_f_-4E-BP1 and _JF585_Halo-eIF4E diffused as fast as the mRNA unbound nuclear counterpart (Fig. 2f), most likely due to the release of the tagged eIF4E:4E-BP1 complexes from the mRNA. Within 2–3 h of torin-1 treatment, residual _JF585_Halo-eIF4E binding to the mRNA was not detected, as demonstrated by the cytoplasmic autocorrelations overlapping with the nuclear autocorrelations (Supplementary Fig. 4a). At this time, _JF585_Halo-eIF4E molecules had translocated to the nucleus in a complex with _JF646_SNAP_f_-4E-BP1 as demonstrated by FCCS and fluorescent microscopy. An enrichment in _JF646_SNAP_f_-4E-BP1 molecules was observed in the cytoplasm, most likely to prevent the binding of eIF4E molecules to the 5’cap since tagged eIF4E:4E-BP1 complexes are still detected by FCCS (Supplementary Fig. 4b, c).

The release of eIF4E from the 5’cap, in living cells, to inhibit translation seems contradictory to the in vitro cap-pull down assay in which eIF4E constitutively binds to the cap-analogs^15–19^.

### Dynamics of eIF4E:eIF4G binding to the mRNA

**Fig. 3:**
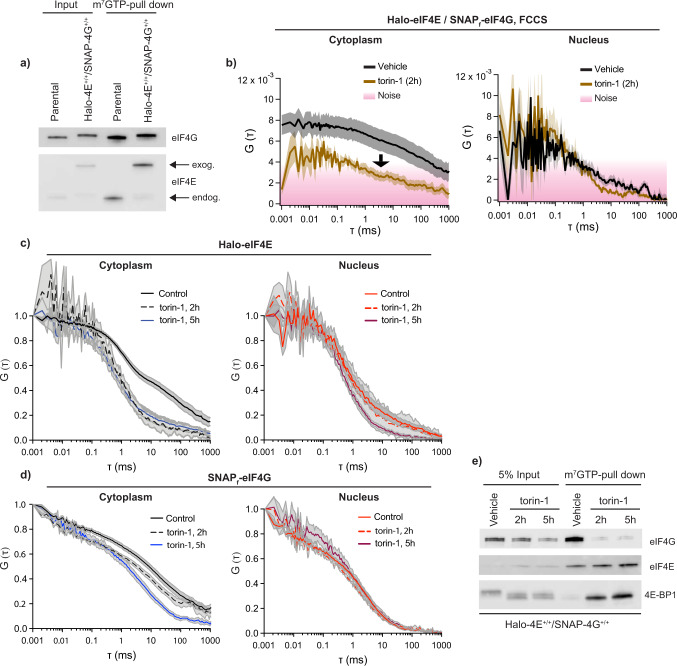
eIF4E and eIF4G binding to the mRNA is detected by FCS upon mTOR inhibition. **a** Parental mESC and double knock-in Halo-eIF4E^+/+^/SNAP-eIF4G^+/+^ total cell lysates (input) were subjected to cap-pull-down assay (m^7^GTP-pull-down) and analyzed by western blotting. eIF4G and eIF4E antibodies showed binding of wild-type and tagged proteins to the 5’cap. Tagging of endogenous eIF4E and eIF4G did not affect cap-binding as compared to the parental counterpart. **b**–**e** Halo-eF4E^+/+^/SNAP-eIF4G^+/+^ cells were treated with vehicle (DMSO) or 250 nM torin-1 for 2 h and 5 h respectively. **b** Simultaneous diffusion of _JF585_Halo-eIF4E and _JF646_SNAP_f_-eIF4G was analyzed by dual color cross-correlation spectroscopy (FCCS) in the indicated conditions. Cross-correlation was detected in the cytoplasm of control cells (*left panel*), but not in the nucleus (*right panel*). Minor residual eIF4E:eIF4G was detected in the cytoplasm upon 2 h mTOR inhibition (*left panel*). **c**, **d** Averaged autocorrelation curves representing individual diffusion of _JF585_Halo-eIF4E (**c**) and _JF646_SNAP_f_-eIF4G (**d**) in the indicated conditions (*N* = 10 ± SEM). In control cells, both _JF585_Halo-eIF4E (**c**) and _JF646_SNAP_f_-eIF4G (**d**) autocorrelations showed slower diffusion as compared to their nuclear counterparts. No changes were detected in the nuclear counterparts. Cytoplasmic eIF4E molecules diffuse as fast as the nuclear counterpart as early as 2 h upon torin-1 treatment, whereas cytoplasmic eIF4G mirrors the nuclear diffusion only after 5 h. **e** Total lysates (input) of cells described in (**c**–**e**) were subjected to cap-pull-down assay (m^7^GTP pull-down) and analyzed by western blotting with the indicated antibodies. eIF4E:eIF4G dissociation occurred as early as 2 h upon torin-1 treatment.

**Fig. 4:**
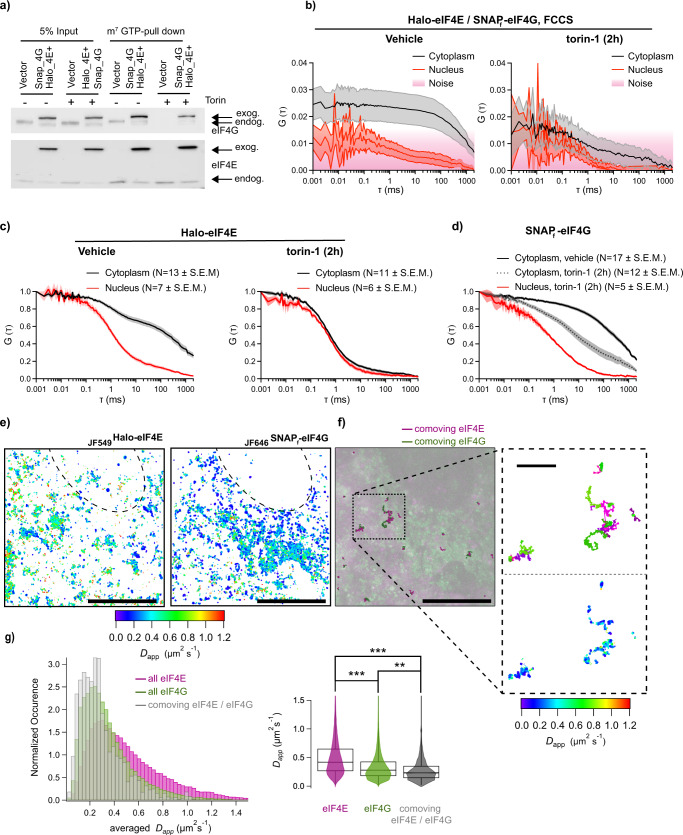
eIF4E and eIF4G binding dynamics detected by FCS and SPT. **a** NIH3T3 cells that express vector control or Halo-eIF4E and SNAP_f_-eIF4G were treated with DMSO (vehicle) or 250 nM torin-1 for 2 h. Total cell lysates (input) were subjected to cap-pull-down assay (m^7^GTP-pull-down) and analyzed by western blotting. eIF4G and eIF4E antibodies detected both endogenous (endog.) and exogenous (exog.) proteins as indicated by the arrows. **b** Simultaneous diffusion of _JF585_Halo-eIF4E and _JF646_SNAP_f_-eIF4G analyzed by dual-color fluorescent cross-correlation spectroscopy (FCCS) in cell treated with vehicle (*left panel*) or 250 nM torin-1 for 2 h (torin-1, *right panel*) in the cytoplasm (gray) and in the nucleus (red) (*N* = 10 ± SEM). Cross-correlation was detected in the cytoplasm of translating cells, but not in the nucleus, and abolished 2 h upon mTOR inhibition. **c**, **d** Individual _JF585_Halo-eIF4E (**c**) and _JF646_SNAP_f_-eIF4G (**d**) averaged autocorrelation curves from (**b**). **e** Simultaneous single-particle tracking of _JF549_Halo-eIF4E and _JF646_SNAP_f_-eIF4G was simultaneously recorded in the cytoplasm of an NIH3T3 (dotted line outlined the nucleus). Left: Diffusion properties of 4319 trajectories of _JF549_Halo-eIF4E and 4001 trajectories of _JF646_SNAP_f_-eIF4G are displayed via heat maps. Each point is false‐colored according to the mean square displacement calculated over all displacements originating in a circle (*r* = 80 nm). **f** Co-movement analysis of _JF549_Halo-eIF4E and _JF646_SNAP_f_-eIF4G. Maximum intensity projections of 5000 frames of _JF549_Halo-eIF4E (in magenta) and 5000 frames of _JF646_SNAP_f_-eIF4G (in green) were simultaneously acquired at 100 Hz. The co-moving _JF549_Halo-eIF4E (bold, magenta) and _JF646_SNAP_f_-eIF4G (bold, green) trajectories are displayed on top. Scale bar: 10 μm. Inset top: co-moving _JF549_Halo-eIF4E/_JF646_SNAP_f_-eIF4G trajectories with their associated diffusion heat maps displayed at higher magnification (scale bar: 1 μm). **g** (left) The distribution of apparent diffusion coefficients shifts to a slower population when _JF549_Halo-eIF4E is comoving with _JF646_SNAP_f_-eIF4G (in gray). (right) Violin plots of one-step mean square displacements for eIF4E (355,730, in magenta), for eIF4G (462,072, in green), and for co-moving eIF4E/eIF4G pairs (7947, in gray). Box quartile method: Tukey. The median line is shown, whisker method: min and max data. Two-sided *t*-test with two mean values of two distributions (*** means *p*  <  0.001, ** means *p*  <  0.01).

FCS and FCCS were able to detect real-time binding and unbinding of _JF585_Halo-eIF4E to the 5’cap mediated by the inhibitory molecule _JF646_SNAP_f_-4E-BP1. If these events occurred on the mRNA, FCS should be capable of detecting changes in the eIF4F complex upon acute mTOR inhibition. Halo and SNAP_f_ tags had been inserted into the *Eif4e* and *Eif4g* loci, respectively, at the N-terminus as described above. The SNAP_f_ tag was fused to the N-terminus of eIF4G to preserve the C-terminus which contains the MAPK-interacting kinases 1 (Mnk-1)-binding site^20^. Mnk1 is recruited on the eIF4F complex by eIF4G to phosphorylate eIF4E at Serine 209. Since eIF4E phosphorylation stimulated the translation of specific mRNAs^21^, perturbing this regulatory step was avoided. Tagging of endogenous eIF4E and eIF4G did not perturb their binding to the 5’cap as compared to the endogenous counterparts lacking the Halo and SNAP_f_ tags respectively (Fig. 3a). The simultaneous diffusion of _JF646_SNAP_f_-eIF4G and _JF585_Halo-eIF4E by FCCS in the cytoplasm and in the nucleus was assessed. FCCS detected stable tagged eIF4E:eIF4G interactions in the cytoplasm, where mRNA translation occurs, but not in the nucleus (Fig. 3b, left panel). Importantly, their interactions were perturbed in the cytoplasm upon mTOR inhibition (Fig. 3b, right panel). Autocorrelation analysis revealed further insights into their sequential binding dynamics. In control cells, both _JF585_Halo-eIF4E and _JF646_SNAP_f_-eIF4G cytoplasmic autocorrelations show that they diffuse as two-components, fast (~11 ms, *D* = 1.53 μm^2^/s) and slow (~923 ms, mRNA bound, *D* = 0.02 μm^2^/s), with the majority of the eIF4G molecules moving on average slower than eIF4E (Fig. 3c, d left panel). In the nucleus, only one-fast component (diffusing at ~3 ms, *D* = 5.59 μm^2^/s) and so free diffusing molecules were detected for both molecules (Fig. 3c, d right panel). Upon 2 h of mTOR inhibition, _JF585_Halo-eIF4E was released from the mRNA (Fig. 3c, left panel), as previously observed in the double knock-in Halo-eIF4E/SNAP_f_-4E-BP1. This coincided with a drop in the polysome levels accompanied by monosome accumulation, consistent with defects in translation initiation (Supplementary Fig. 5a). Unlike eIF4E, major changes in the diffusion of cytoplasmic _JF646_SNAP_f_-eIF4G were observed only after five hours of mTOR inhibition (Fig. 3d, left panel). After prolonged mTOR inhibition, cytoplasmic _JF646_SNAP_f_-eIF4G autocorrelations mirrored the nuclear fast component (Fig. 3d), suggesting that eIF4G resided longer than eIF4E on the mRNA. Since eIF4E enables eIF4G interactions near the 5’cap to initiate translation this result was unexpected. eIF4G can also interact with the mRNA via two RNA binding domains in the protein, via the helicase eIF4A and the poly(A)-binding protein (PABP). The long association of eIF4G on mRNA could be due to these multiple means to bind mRNA. Fusion of the SNAP_f_ and Halo tags to eIF4G and eIF4E, respectively, could impair eIF4F complex formation. In vitro cap-pull-down assays demonstrated that tagged eIF4E:eIF4G interactions remain mTOR-dependent (Fig. 3e), suggesting that the unexpected kinetics observed in living cells are not caused by the presence of the genetically encoded tags. To further rule out the possibility that tagging of two initiation factors altered their temporal interactions in live cells, FCS was performed in Halo-eIF4E or SNAP_f_-eIF4G knock-in differentiated mESCs (Supplementary Fig. 6a–c). The same binding dynamics were observed in these cells, upon mTOR inhibition eIF4G also bound longer to the mRNAs compared to eIF4E (Supplementary Fig. 6d, e). The apparent concentration of initiation factors was determined in order to rule out artefacts due to the high concentration of initiation factors. The apparent concentration was estimated to be 249 nM for _JF585_Halo-eIF4E and 113 nM for _JF646_SNAP_f_-eIF4G, therefore values are well suited for FCS^22,23^ (Supplementary Fig. 7). In order to investigate whether these unexpected binding dynamics were unique to differentiated mESCs, SNAP_f_-eIF4G and Halo-eIF4E were expressed in NIH3T3 cells (Fig. 4a). Dual color FCCS was able to detect stable eIF4E:eIF4G interactions in the cytoplasm, but not in the nucleus (Fig.4b, left panel). Acute mTOR inhibition abolished most of the eIF4E:eIF4G interactions as previously observed in differentiated mESCs (Fig. 4b, right panel). Individual autocorrelations confirmed that _JF585_Halo-eIF4E, unlike _JF646_SNAP_f_-eIF4G, was rapidly released from the mRNA (Fig. 4c, d) to retard translation initiation as observed by polysome profiling (Supplementary Fig. 5b).

In order to validate these findings with an orthogonal approach, single particle tracking (SPT) was used to investigate the differential diffusion of initiation factors. SPT revealed that _JF646_SNAP_f_-eIF4G molecules diffused on average more slowly than _JF585_Halo-eIF4E (Fig. 4e, f and Supplementary Movie 1) as determined using FCS. The tagged eIF4G apparent diffusion coefficient ranged between 0.1 and 0.8 μm^2^/s. Interestingly, the diffusion coefficient for the free and mRNA-bound large ribosomal subunits is 0.1 and 0.4 μm^2^/s, respectively^24^. The slower diffusion of eIF4G compared to eIF4E could be due to association with the small ribosomal subunit through eIF3^25^ when the ribosome was not bound to the mRNA. Individual trajectories for _JF585_Halo-eIF4E and _JF646_SNAP_f_-eIF4G revealed that the eIF4G apparent diffusion coefficient further decreased when co-moving with eIF4E, most likely due to their stable interactions with the mRNA to initiate translation (Fig. 4g histogram and violin plot). Altogether, these data revealed that FCS and SPT could detect the binding of initiation factors to the mRNA to initiate cap-dependent translation in living cells.

### Real-time detection of cap-dependent translation “hot-spots” in primary neurons

Local mRNA translation has been postulated to play a critical role in neuronal function, including neurite remodeling, synapse formation and pruning, and synaptic plasticity^26^. Upon their transcription, mRNAs were transported into the neuronal processes in a translationally repressed state. During transport, most of the mRNA binding proteins (RBPs) prevent the assembly of the eIF4F complex by blocking eIF4E binding to the 5’cap^27–29^. As demonstrated above, differential diffusion of initiation factors was a read-out for localized events of cap-dependent translation initiation.

**Fig. 5:**
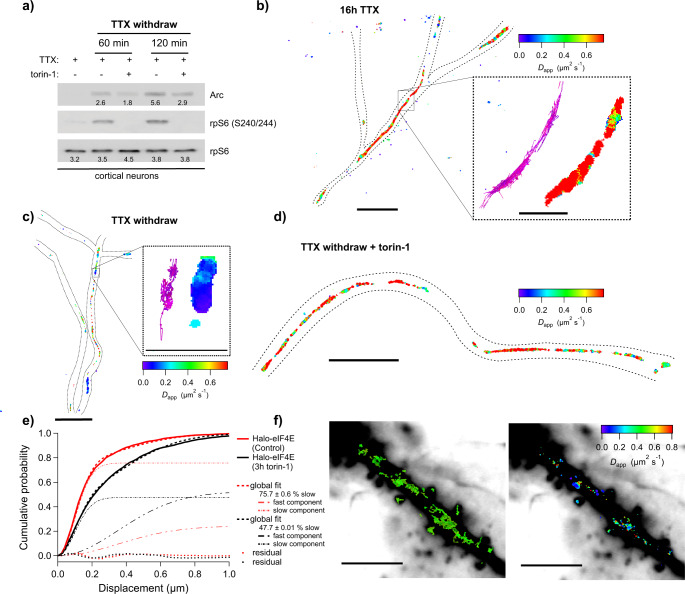
Single-particle tracking of Halo-eIF4E in primary hippocampal neurons. Rat hippocampal neurons were activated with TTX withdrawal with or without 250 nM torin-1. **a** Total cell lysates were analyzed by western blotting with the antibodies indicated next to the corresponding blot (*n* = 2). **b** Diffusion heat map of Halo-eIF4E trajectories obtained in inactivated neurons (16 h TTX) at a frame rate of 100 Hz (with a dotted neuronal outline, scale bar: 5 μm. Each point in the image is false‐colored according to the mean square displacement calculated over all displacements originating in a circle (*r* = 80 nm). Inset: Trajectories and corresponding diffusion heat map (scale bar: 1 μm). **c** Diffusion map of Halo-eIF4E of activated neurons after TTX withdrawal (scale bar: 5 μm). Inset: Trajectories and corresponding diffusion heat map (scale bar: 1 μm). **d** Diffusion speed of Halo-eIF4E in neurons treated with 250 nM torin-1 (red colors) (scale bar: 5 μm). **e** Single particle tracking was recorded at a frame rate of 200 Hz. The cumulative distribution function (CDF) of all single-molecule displacements for Halo-eIF4E is shifted to the left when compared to neurons treated with torin-1, which indicates a shift towards slower diffusion for Halo-eIF4E undergoing cap-dependent translation. CDF was obtained from 1226 and 742 trajectories in 10 individual dendrites of neurons treated with vehicle control or torin-1, respectively. The global two-component fit returns a global apparent *D*_slow_ of 0.32 ± 0.006 µm^2^/s and a global apparent *D*_fast_ of 4.10 ± 0.076 µm^2^/s. While the majority of control Halo-eIF4E exhibit slow diffusion (76 ± 0.6%), this percentage drops to 48 ± 0.6% when treated with torin-1. The data was recorded at 200 Hz, and we report apparent diffusion coefficients for that frame rate. **f** Left: Medium-intensity projection of 10,000 frames of _JF549_Halo-eIF4E recorded simultaneously with sparsely photo-activated _PA-JF646_Halo-eIF4E molecules (individual _PA-JF646_Halo-eIF4E trajectories are shown in green) at 56 Hz frame rate (scale bar: 10 μm). Right: The associated _PA-JF646_Halo-eIF4E diffusion heat map depicts that slow Halo-eIF4E molecules (in blue) linger near what appear to be spines in activated neurons (scale bar: 10 μm). Spines are indicated with an asterisk.

In order to detect cap-dependent translation in neuronal processes, Halo-eIF4E was expressed in primary neurons at a similar concentration as the endogenous counterpart (Supplementary Fig. 8) and its diffusion was analyzed upon global neuronal activation by tetrodotoxin (TTX) withdrawal. This method utilized prolonged treatment of cultured neurons with TTX, a sodium channel blocker, followed by its washout to trigger neuronal activity. TTX withdrawal stimulated mTOR activity within the first two hours (Fig. 5a). Under this activation protocol, a significant portion of _JF646_Halo-eIF4E molecules diffused slower in the dendrites (Fig. 5c; Supplementary Movie 3), in contrast to inactivated neurons where the majority of the tagged eIF4E molecules diffused freely in the dendrites (Fig. 5b; Supplementary Movie 2) or upon acute mTOR inhibition (Fig. 5d; Supplementary Movie 4). _JF646_Halo-eIF4E movements in activated neurons best fit with two components: a fast apparent diffusion coefficient of 4.10 μm^2^/s, and a slower one of 0.32 μm^2^/s. Translating mRNAs had an apparent diffusion coefficient of ~0.1 μm^2^/s^24^. This suggested that the slow-moving eIF4E may reflect early events of translation initiation. Indeed, the two-component fit of _JF646_Halo-eIF4E trajectories upon mTOR inhibition revealed a reduction in the slower diffusion component (from 76% to 48%) (Fig. 5e).

**Fig. 6:**
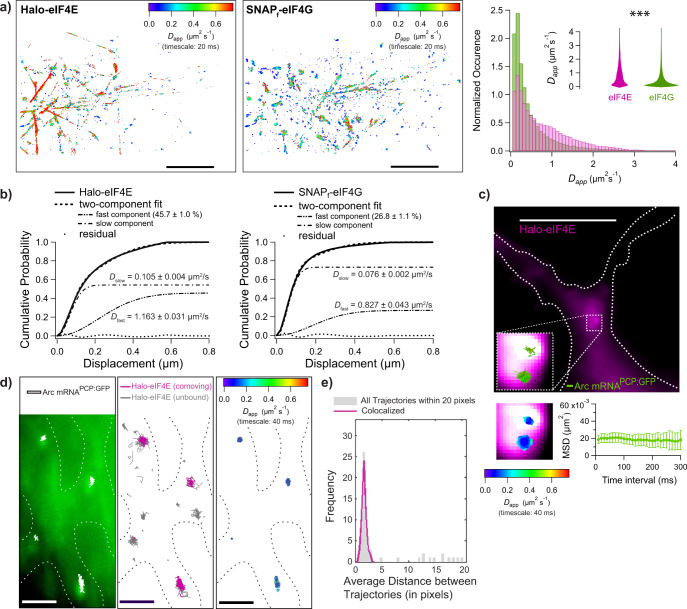
Simultaneous single-particle tracking of endogenous Halo-eIF4E and SNAP_f_-eIF4G in mESC cells that were differentiated into neurons. **a** Top left: Diffusion heat map of 18,220 _JF585_Halo-eIF4E trajectories from eight 10,000 frames 50 Hz movies obtained from a dense network consisting of more than 100 individual dendritic branches (scale bar: 10 μm). Top center: The corresponding heat map of the simultaneously recorded 26,438 _JF646_SNAP_f_-eIF4G trajectories. Top right: The distributions of apparent diffusion coefficients of _JF585_Halo-eIF4E (in green) and _JF646_SNAP_f_-eIF4G (in magenta). Two-sided *t*-test with two mean values of two distributions (*** means *p*  <  0.001). **b** Cumulative distribution functions (CDF) were obtained from 26,438 eIF4G and 18,220 eIF4E trajectories. While the majority of Halo-eIF4E exhibit slow diffusion (54.3 ± 1.0%), this percentage increases to 73.2 ± 1.1% for SNAP_f_-eIF4G. **c** Thick dendritic process of a cortical neuron from ARC^P/+^;PCP-GFP;v-Glut2Cre mice expressing Halo-eIF4E after TTX withdrawal (dotted outline, scale bar: 10 μm). The median projection of the _JF646_Halo-eIF4E channel of 5000 frames was recorded at 100 Hz (in magenta). Overlay of two ARC mRNA trajectories (in green) labeled with PCP-GFP that were simultaneously recorded. 1.6 × 1.6 μm^2^ insets of _JF646_Halo-eIF4E and two ARC mRNA trajectories with their associated diffusion heat maps. The mean square displacement (MSD) curve of the two ARC mRNA trajectories depicts corralling of mRNAs and reveals an exploration area of 0.02 μm^2^. **d** Dendritic branches of cortical neurons from ARC^P/P^ mice expressing Halo-eIF4E after TTX withdrawal (dotted outline, scale bar: 2 μm). Left: Median projection of the PCP-GFP channel of 6000 frames recorded at 100 Hz outlines a dense region of neuronal processes. Overlaid are the trajectories of four ARC mRNA molecules (in white). Middle: Co-moving Halo-eIF4E molecules (in magenta) and unbound Halo-eIF4E molecules are depicted in gray. Right: The diffusion heat map of co-moving mRNA/ Halo-eIF4E molecules depicts almost static movements of the translation factor interacting with ARC-mRNA. **e** The distribution of distances of all ARC-mRNA and all eIF4E particles (in gray) consists of a peak of short values (<4 pixels) above a flat baseline. The co-movement algorithm (magenta line) efficiently selects the peak of colocalized trajectories.

Local mRNA translation at activated synapses is thought to be associated with long-lasting synaptic plasticity^26^. In mature dendrites, we detected clusters of single exogenous _JF646_Halo-eIF4E molecules lingering near the spines and diffusing slowly at or <0.1 μm^2^/s (Fig. 5f; Supplementary Movie 5), suggesting that translation initiation occurred in these areas. In order to avoid artifacts due to overexpression of initiation factors, early events of endogenous eIF4F complex assembly were interrogated. The double-knock in Halo-eIF4E and SNAP_f_-eIF4G mESC were differentiated into neurons^30^ (Supplementary Fig. 9a). As with primary neurons (Fig. 5a), TTX withdrawal activated mTOR kinase and eIF4E (Supplementary Fig. 9b). In activated neurons, _JF549_Halo-eIF4E and _JF646_SNAP_f_-eIF4G had differential diffusion properties as previously detected by FCS and SPT in fibroblasts (Fig. 6a; Supplementary Movie 6). eIF4E and eIF4G diffusions were the best fit with two components, with the faster and slower components having apparent diffusion coefficients of >1 and 0.1 μm^2^/s, respectively (Fig. 6b). Since the data was recorded at a 50 Hz, the frame rate is not fast enough to record true microscopic diffusion coefficients, and we report apparent coefficients (see Fig. 6b). These data were consistent with eIF4F complex formation to initiate translation in dendrites.

Using SPT, the slower diffusing Halo-eIF4E molecules were tested as to whether they bound mRNA. ARC is an immediate early gene rapidly transcribed upon neuronal activation to consolidate long-term memory formation^31^. eIF4E activity was shown to be rate-limiting for ARC translation^32^ and mTOR inhibition attenuated Arc protein expression without affecting mRNA stability (Fig. 5a and Supplementary Fig. 9c). In order to label the endogenous ARC mRNA, a transgenic mouse was generated that constitutively expressed GFP fused to the coat protein PCP (PCP-GFP). The PCP-GFP tagged the endogenous ARC mRNA in which a tandem array of PP7 binding sites (PBS) had been inserted into the 3’UTR of the ARC gene (*Arg 3.1*)^33^. Exogenous Halo-eIF4E was expressed in primary neuronal cultures isolated from the mice. ARC mRNA had been previously shown to be transported along the dendrites, with the anterograde and retrograde transport often interrupted by pauses^33^. However, it remained unclear whether the stationary mRNA was actively translated. In a dendrite, a dense area was observed enriched with _JF646_Halo-eIF4E molecules (Fig. 6c; Supplementary Movie 7). Importantly, two stationary ARC mRNA molecules were detected inside this dense area (Fig. 6c; Supplementary Movie 7). The binding of _JF646_Halo-eIF4E molecules to the ARC mRNAs was imaged with single-molecule precision and both fast and slow-moving eIF4E molecules were tracked (Fig. 6d; Supplementary Movie 8). ARC mRNA trajectories were spatially and temporally correlated with _JF646_Halo-eIF4E trajectories to identify a co-moving population for diffusion analysis. Specific co-moving events are dominant which results in a large enrichment in short distances between ARC mRNAs and tagged eIF4E (Fig. 6e, almost all trajectories are under a red curve). The trajectories were used to generate a diffusion map and demonstrated that mRNA:Halo-eIF4E molecules were almost immobile. This demonstrated that the static translation factors likely represented translation initiation in neuronal processes.

## Discussion

Resolving the regulation of fundamental cellular processes with sub-cellular and single-molecule resolution in living cells is necessary to elucidate how cellular homeostasis is preserved. mRNA translation is a tightly regulated process, where dysregulation leads to several pathological conditions^34^. How this regulation occurs is not completely understood, therefore inhibiting the development of effective therapies. Regulation of translation presents a challenge for any live-cell imaging methodology due to its broad range in concentration (from a single mRNA to hundreds of thousands of ribosomes and perhaps millions of translation factors per cell), as well as the broad temporal dynamic range (from milliseconds of binding and unbinding of translation factors, to minutes-long translation times). For this reason, several live-cell imaging methodologies have been developed in order to visualize hot spots of mRNA translation^35–38^, but they rely on a specifically fluorescent mRNA and/or simultaneous labeling of nascent polypeptides from a specific mRNA. We have previously applied dual-color FFS brightness analysis to detect the binding of a translation inhibitory protein to mRNA during steady-state, but this lacked temporal and spatial resolution^39^. To date, methodologies able to elucidate the dynamic assembly of individual translation complexes on their target mRNAs in response to cues are lacking. In this work, live-cell spectroscopy was able to resolve the diffusion and binding properties of thousands of molecules in tens of seconds and, when combined with an orthogonal approach using SPT, was able to reveal sites of global translation. The approach was then applied to interrogate the interactions between the initiation factor eIF4E and a physiologically important mRNA in neurons.

Furthermore, our approach revealed details on the binding stoichiometry of initiation factors to the mRNA. Live imaging demonstrated that half of the endogenously labeled cap-binding proteins eIF4E and eIF4G were bound to mRNA, with the remaining molecules freely diffusing. Previous work demonstrated that a reduction in eIF4E levels by 50% did not affect mouse development^7^. Our data support the hypothesis that a surplus of eIF4E exists. We speculate that the cells benefit from an excess of eIF4E by ensuring homogeneous distribution throughout the cytoplasm to rapidly activate bursts of translation, for instance, increased mitochondria activity^16,40^ or cell proliferation^19,41^. Live imaging revealed additional insights into the regulatory dynamics of translation. In living cells, eIF4E binding to 4E-BP1 triggered the release of eIF4E from the 5’cap within 30 min upon mTOR inhibition, while eIF4G resided longer on the mRNA to reinitiate translation. The release of eIF4E from the mRNA in living cells was unexpected since in a cap-pull-down assay eIF4E constitutively binds the cap analog, even when bound to 4E-BPs. This may be due to the difference between the two methodologies. The cap-binding assay relies on a cap-analog that has a very high affinity for eIF4E and lacks any regulatory sequences that may be present on target mRNAs in living cells. In the native environment, molecular behavior is analyzed (i) on the target mRNAs, thereby preserving any potential regulatory sequences and (ii) in presence of molecules that could facilitate eIF4E dissociation from the 5’cap, for example, LARP1^42^.

eIF4E is localized in the cytoplasm and in the nucleus, with the nuclear eIF4E immunostaining either enriched in discrete nuclear bodies^43^ or diffuse^44^. Several non-canonical nuclear functions have been attributed to eIF4E^45^ to ultimately regulate the export of its target mRNAs into the cytoplasm^46^. In the cell types we have analyzed (NIH3T3 fibroblasts, primary neurons, and mESC-derived fibroblasts and neurons) eIF4E spherical bodies were not detected and, in agreement with that, temporal autocorrelations obtained by FCS demonstrated that the majority of the eIF4E molecules inside the nucleus diffuse as one fast-component and are not bound to its target mRNAs. In agreement, prolonged mTOR inhibition (>3 h), led to the accumulation of endogenous eIF4E:4E-BP1 complexes in the nucleus most likely to prevent eIF4E:eIF4G interactions and stable binding of eIF4E to the 5’cap. Our data demonstrated that elucidating molecular interactions in the native environment, while minimally perturbing protein function and stoichiometry, was vital to characterize the regulation of fundamental cellular processes.

Single-molecule detection of mRNAs revealed differential distributions in subcellular compartments in neurons, fibroblasts, and epithelial cells emphasizing that locally regulated translation synthesizes proteins where needed^47^. Regulation of mRNA translation is heterogenous, mRNAs are differentially translated based on their structure, post-transcriptional modifications, and mRNA sequence^48^. The diversity of translation factors and ribosomes may fine-tune the translation of individual mRNAs in subcellular compartments^49^. This can be resolved by detecting the binding of translation complexes on specific mRNAs in living cells.

By generating a diffusion map of endogenous initiation factors, we were able to detect slow diffusion of eIF4E (*D*_app_ < 0.1 μm^2^/s) consistent with its binding to the mRNA in dendrites of activated primary and mESC-derived neurons and in the proximity of the spines. This apparent diffusion coefficient of bound eIF4E is consistent with the microscopic diffusion coefficient we obtained from FCS (see Fig. 1a, with a residence time of 372.7 ms for bound eIF4E corresponding to *D*_microscopic_ = 0.05 μm^2^/s). The mechanism behind this slow diffusion of eIF4E was confirmed by its similarly slower diffusion properties in co-movement with ARC mRNA. Binding events of endogenous eIF4E:eIF4G were also detected in the dendrites of activated neurons. Notably, in neurons as in fibroblasts, we observed an overall differential diffusion with free eIF4G molecules diffusing more slowly than unbound eIF4E (*D*_app_ = 1.29 and 1.82 μm^2^/s, respectively, see Fig. 6b), suggesting that it remains bound to the mRNA after initiation. Interestingly, ARC expression is not completely inhibited at 120 min upon torin-1 treatment (Fig. 5a). This is consistent with the finding that in living cells most eIF4G molecules remain on the mRNA two hours upon torin-1 treatment, as demonstrated by minimal changes in its diffusion as compared to translating cells (Fig. 3d). All together, these data suggests that eIF4G alone may be sufficient to promote re-initiation. It is important to note that when recording movies of rapid cellular diffusion, the apparent diffusion coefficients obtained at a camera frame rate of 50 Hz do not correspond to microscopic diffusion coefficients (as obtained from FCS in Fig. 1a, where free eIF4E has a residence time of 1.14 ms, which corresponds to *D*_microscopic_ = 14.78 μm^2^/s).

The role of cap-dependent translation in neuronal physiology was demonstrated using genetics and pharmacological approaches^50^, but the role of spatially localized translation in neuronal processes has remained unclear since these approaches lacked sufficient spatial and temporal resolution. Our methodology identified early events of cap-dependent translation with the single-mRNA resolution, revealing that neuronal translation occurs in clusters, recently reported in living Drosophila embryos as well^51^. Translational hot spots are reminiscent of transcriptional “hot spots”^52^ and may indicate a common mechanism to enhance the concentration of factors in local regions of the cell, perhaps through interactions of disordered domains.

Aberrant mRNA translation results in a broad spectrum of diseases, including cancer^53^, metabolic and neurodegenerative disorders^34^, as well as viral infection^54^. Targeting the translation machinery in diseases is challenged by our limited understanding of this paramount process. This approach will have important implications in understanding how translation dysregulation leads to pathological conditions and identifying new therapeutic targets. Importantly, the approach we described here can elucidate fundamental cellular processes without perturbing the native environment when they result in a measurable shift in diffusion.

## Methods

### Cell lines, neuronal cultures, and compounds

NIH3T3, obtained from America Type Culture Collection (ATCC) was maintained in phenol red-free Dulbecco’s modified Eagle medium supplemented with 10% fetal bovine serum, 1% antibiotic–antimycotic mix (ThermoFisher), and 1% GlutaMAX™ (ThermoFisher). JM8.N4 mouse ESCs from the C57BL/6N (a generous gift from Liu laboratory, Janelia Research Campus) were maintained in Knockout DMEM (GIBCO #10829-018) supplemented with 10% fetal bovine serum ES cell qualified (ATCC® SCRR-30-2020™), 1X GlutaMAX™ Supplement 100X (GIBCO, #35050-061), 1X MEM non-essential amino acids solution, 100X (GIBCO, #11140050), 0.1 mM 2-mercaptoethanol (GIBCO, Cat#21985-023), 1000 U/mL LIF (EMD Millipore, # ESG1106), 1μM PD03259010 (Millipore Sigma, #PZ0162), 3μM CHIR99021 (StemCell Technologies, #72052). The medium was renewed every second day. All cell lines were maintained at 5% CO_2_ at 37 °C.

Primary rodent work was performed in accordance with protocols approved by the Janelia Research Campus Institutional Animal Care and Use Committee (IACUC) guidelines. Mice were housed in a 12 h light/dark cycle. Newborn pups were euthanized, and cortical tissue dissociated, in papain enzyme (Worthington Biochemicals, ~25 U/cortical pair) in dissection solution (10 mM HEPES pH 7.4 in Hanks’ balanced salt solution) for 30 min at 37 °C. Following trituration and filter through a 40 μm strainer, single cells were seeded in Plating media [28 mM glucose (Acros #41095), 2.4 mM NaHCO_3_ (Sigma-Aldrich S-8875), 100 µg/mL transferrin (Calbiochem #616420), 25 µg/mL insulin (Sigma #I-6634 or #I-1882), 2 mM l-glutamine (GIBCO #25030-081), 100 U/mL penicillin, 10 µg/mL streptomycin and 10% fetal bovine serum (heat inactivated; HyClone)] in MEM (GIBCO 51200-038) and NbActiv media (BrainBits, LCC) mixed in a 1:1 ratio. 16 h later, the media was replaced with Plating media and NbActiv mixed in a 1:33 ratio.

Rat cortical cultures were seeded on poly-d-lysine hydrobromide (PDL) (Sigma) diluted in sterile water at 0.2 µg/mL. Cultures were then maintained by feeding cells twice a week by replacing old media 1:1 with fresh NbActiv media.

Newborn mouse neuronal cultures were plated on PDL and laminin (5 μg/mL)-coated plates. Mouse neurons were seeded in Plating media [B27/Neurobasal media, 25 μM Glutamax and 5% FBS to mouse Neuron culture media (same as mouse Plating media without FBS in the ratio of 1:1. (Gibco #17504044).

Where indicated, cells were treated with 250 nM torin-1 (Tocris) dissolved in DMSO or with an equal amount of DMSO as vehicle control. Neuronal cultures were treated with 1.5 μM Tetrodotoxin TTX) (Sigma-Aldrich, #554412). After 16 h, TTX was removed and substituted with conditional media with vehicle control or 250 nM torin-1 upon 3 extensive washes with Neuronal Buffer provided by the Janelia Research Campus Media Facility.

### Differentiation of mESC to neurons or fibroblasts

For neuronal differentiation, a single mESC cell suspension was obtained and resuspended in embryoid body (EB) media [Knockout DMEM (GIBCO, 10829-018), 10% FBS ES Cell Qualified (ATCC® SCRR-30-2020™), 1X GlutaMAX™ (GIBCO, #35050-061), 1X MEM non-essential amino acids solution (GIBCO, Cat#11140050) and 55 μM 2-mercaptoethanol]. 50 cells/well were plated and EBs grow in AggreWell™400 dish (StemCell Technologies #07010), according to the manufacturer’s instructions. Briefly, EBs were maintained for 8 days in EB media in 7% CO_2_ at 37 °C. At day 4, EB media was supplemented with 5 μM retinoic acid (Sigma, #R2625). The medium was replaced every 48 h. On day 8, EBs were dissociated and filtered through a cell strainer (Falcon, #352340). Single-cell suspension was resuspended in BrainPhys complete media and 1.13 × 10^5^ cells/cm^2^ were seeded in plates coated with 15 μg/mL poly-l-ornithine (Sigma #P4957) and 0.5 μg/cm^2^ laminin (Gibco #23017-015). Cells were incubated for 2 h in 7% CO_2_ at 37 °C. Media was replaced with fresh BrainPhys complete media after 2, 24, and 48 h. Brainphys Neuronal Medium (#05790) was supplemented with 2% NeuroCult SM1 neuronal supplement (#05711), 1% N2 Supplement-A (#07152), 20 ng/mL BDNF (#78005), 20 ng/mL GDNF (#78058), 1 mM Dibutyryl-cAMP (#73882), 200 nM l-ascorbic acid (Millipore, AX1775-3). All supplements were from Stemcell Technologies unless otherwise specified. Differentiating mESC was maintained in 5% CO_2_ at 37 °C. After 48 h, neurons were fed by replacing half of the media every 5 days.

For fibroblast differentiation, mESC were seeded at low density (~20%) in 10 μg/mL fibronectin-coated plates in mESC complete media for 16 h. mESC were then cultured in absence of LIF and 2i (PD03259010 and CHIR99021) for 48 h to induce fibroblast differentiation.

### Lentiviral shRNA, plasmids, Crispr/Cas9 knock-in constructs, and generation of cell lines expressing tagged translation factors

Non-targeted shRNA control (Scrambled, SHC216) and the EIF4E shRNA (TRCN0000077477), 4EBP1 shRNA (TRCN0000335449), and EIF4G1 shRNA (TRCN0000096812) targeting the coding sequence (CDS) were all from Sigma. Lentiviral backbone pLV-EF1a-IRES-Neo was a gift from Tobias Meyer (Addgene plasmid #85139). Mouse eIF4E, 4E-BP1, and eIF4G1 CDS were synthesized (GeneScript Biotech) by fusing a spacer AGC-GGC-GGA-GGC-GGA-TCC- GGC-GGA-GGC-GGA-AGC (Ser-Gly-Gly- Gly-Gly-Ser- Gly-Gly- Gly-Gly-Ser) at the N-terminal. Each CDS was then subcloned into pLV-EF1a-IRES-Neo. Halo or SNAP-tag was then inserted upstream of the spacer. To generate an shRNA-insensitive CDS, silent mutations were inserted inside the seed sequence targeted by the shRNA. Viral supernatant for each of the indicated constructs was generated by the Janelia Viral Tool facility. Infection was carried out with 8 μg/mL polybrene. 48 h later, NIH3T3 was selected with 500 μg/mL G418 for 7 days, at the end of which, protein expression was analyzed by western blotting. The endogenous counterparts were then silenced by infecting the cells with lentiviral particles carrying shRNA or Scrambled control respectively, as described above. The selection was performed with 5 μg/mL puromycin. Protein expression was analyzed by western blotting and the cells were maintained in complete DMEM with 500 μg/mL G418 + 5 μg/mL puromycin. To generate NIH3T3 that expressed both Halo-eIF4E and SNAP_f_-eIF4G, cells that expressed SNAP_f_-eIF4G were overlayed with Halo-eIF4E viral supernatant as described above. 48 h later, cells were stained with 100 nM JF_646_-Halo ligand for 30 minutes, washed three times in 1× PBS and sorted.

To generate knock-in mESC, guide RNAs were cloned into pTij-U6-sgRNA-CBh-Cas9-PGK-puroR (provided by the Liu lab, Janelia). Three different gRNAs were designed and experimentally tested for each gene (*Eif4e*: ENSMUST00000029803.11; *Eif4g1*: ENSMUST00000115460.7; *Eif4epb1* ENSMUST00000033880.6). The following were able to generate homozygote knock-in:

eIF4E #3 − 5’ GAACCGGTGAGTATTGCCTT 3’

eIF4G1 #1 − 5’ GTGCTGGGGGGACCCTAATGTGG 3’

4E-BP1 #11.1 – 5’ GCGTGCAGGAGACATGTCGG 3’

The donor sequence (Halo or SNAP_f_ tag flanked by ~700 nucleotides complementary to the targeting sequence) was cloned into pUC19 (provided by the Liu lab, Janelia). 500 μg of gRNA and 500 μg of donor plasmids were electroporated in 1 × 10^6^ mESC using P3 Primary Cell 96-well Nucleofector™ Kit (Lonza, PBP3-22500) according to the manufacturer’s instruction. After 3 days, mESC were stained with 100 nM JF_646_ Halo- or SNAP-tag ligands for 30 min, and single cells were sorted in a 96-well plate. Clones were expanded and genotyped using the following primers, and homozygosity was further validated by western blotting.

eIF4E_Genot_F2: 5’ GTGGACCGGGGACTGGGGAGAC 3’

4E-BP1_Genot_F1: 5’ AGTTCTGCCACCGTCATCCCTACC 3’

eIF4G_Genot_F2: 5’ GCCCCGTGGAGCCAGGTTGATA 3’

### Western blotting and antibodies

Western blotting was performed as previously described^15^. Briefly, cells were washed 3 times in ice-cold 1X PBS and scraped in RIPA lysis buffer [10 mM Tris–HCl pH 8.0, 150 mM NaCl, 1% Triton-X100, 0.5% sodium deoxycholate, 0.1% sodium deoxicholate, 1 mM EDTA, 5 mM NaF, 10 mM β-Glycerophosphate, 1 mM Vanadate (New England BioLabs) and cOmplete™, Mini, EDTA-free Protease Inhibitor Cocktail (Millipore)]. Total protein lysates were separated into 12% or 4–15% Mini-PROTEAN® TGX™ Precast Protein Gels (BioRad), transferred on a nitrocellulose membrane (BioRad), and transferred using a semi-dry apparatus (BioRad). The following antibodies were diluted in [1X Tris Buffered Saline solution (TBS) pH 7.4 (ThermoFisher), 5% bovine serum albumin (Sigma-Aldrich) and 0.1% Tween-20]: anti-eIF4E (monoclonal, BD Transduction Laboratories™ #610269), anti-4E-BP1 (#9644), anti-eIF4G1 (#2498), anti-phosho-4E-BP1 Thr37/46 (#2855), anti-phospho-4E-BP1 Ser65 (#9451), anti-phosho-rpS6 Ser 240/244 (#2215), anti-rpS6 (Santa Cruz Biotechnology, #sc-74459) all from Cell Signaling Technology, anti-GAPDH (#G8795) and anti-b actin (#A5441) from Sigma-Aldrich, anti-ARC (Santa Cruz Biotechnology, #sc-17839, anti-HaloTag (Promega, #G9211), anti-SNAP-tag (New England Biolabs, #P9310S) and anti-Cyclin D1 (BD Bioscience, #556470). Primary antibodies listed above were used at 1:1,000. Secondary antibodies, rabbit (Sigma-Aldrich) and mouse (Amersham) IgG HRP linked, were used at 1:5,000. Signals were revealed by Clarity Western ECL Substrate (BioRad) and detected using a ChemiDoc™ MP Imaging System (BioRad, #12003154).

### Imaging system

Stroboscopic single particle tracking was performed using a custom-built three-camera microscope as described in ref. 55. Briefly, the microscope is equipped with an Olympus ×100 NA 1.5 TIRF objective. The design around the easily accessible RAMM frame (ASI) allowed three separate tube lenses (LAO-300.0, Melles Griot) to be placed close to the objective and into the infinity space of the microscope, resulting in ×166.66 overall magnification. The three Andor iXon Ultra EMCCD cameras were operated simultaneously using Micro-Manager (1.4.20)^56^ (cooled to −80 °C, 17 MHz EM amplifiers, preamp setting 3, Gain 400). The cameras were start-synchronized using a National Instruments DAQ board (NI-DAQ-USB-6363). Lasers (490 nm, 561 nm, 639 nm, all Vortran Stradus lasers) stroboscopically illuminated the sample using peak power densities of ∼1.7 kW/cm^2^ using HiLo illumination. All lasers allow for direct TTL modulation, and stroboscopic excitations are synchronized to the frame times of the respective cameras via LabVIEW 2012 (National Instruments). During imaging, cells were maintained at 37 °C and 5% CO_2_ using a Tokai-hit stage top incubator and objective heater.

### Cellular labeling using Halo-tag or SNAP-tag ligands

Immediately prior to imaging, cells were labeled for single-molecule imaging as described in^57^. The labeling conditions for co-tracking in fibroblasts were 20 nM JF549-SNAP-tag ligand^58^ (30 min) and JF646-Halo tag ligand (0.5 nM, 15 min)^59^. For Halo-eIF4E tracking in neurons, we used 100 nM JF646-Halo tag ligand (15 min), and for co-tracking in neurons we labeled with 100 nM JF646-SNAP and JF549-Halo tag ligands (15 min). For Halo-eIF4E imaging in spines (see Fig. 5f), we also added PA-JF646-Halo tag ligand (15 min, 100 nM)^60^. PA-JF646 was photoconverted by 100-μs-long excitation pulses of 407 nm laser light (Vortran Stradus, 50 W/cm^2^) every second. During image acquisition, the pulse length was increased to 200-μs-long pulses.

### 3-camera in silico registration

Slide-mounted TetraSpeck fluorescent microspheres (T14792, Invitrogen) were imaged before and after data acquisition. Short movies (100 frames) of the fluorescent broadband beads in all three channels were registered in Fiji/ImageJ2 (v.2.3.0/153q) using the descriptor-based series registration (2d/3d + t) Fiji plugin^61^. For more than two-channel bead registration, the frames have to be set as channels (in Fiji: Image > Hyperstacks > Re-order Hyperstack). Sub-pixel accurate registration was achieved via Gaussian mask localization fitting of spots, which were used to compute affine (2D) transformation models^62^. Since unbiased noise distribution is essential for accurate single particle identification and tracking, the Fiji plugin now allows for nearest-neighbor interpolation that does not change actual pixel intensities and thereby preserves the original noise distribution (as compared to linear or other higher-order interpolation schemes). This update is available via the Fiji Updater (http://fiji.sc/Downloads). It can be found under Plugins > Registration > Descriptor based Registration (2d/3d) and Plugins > Registration > Descriptor based Series Registration (2d/3d + t).

### Single particle tracking and analysis

Trajectories were obtained using DiaTrack (v. 3.04, Semasopht), which identifies and fits the intensity spots of fluorescent particles with 2D Gaussian functions matched to the experimentally determined point-spread function. The diffusion maps were created using tracking routines written in IGOR Pro 8.04 (WaveMetrics) as described in ref. 24. Briefly, local apparent diffusion coefficients of eIF4E and eIF4G are calculated and mobility is evaluated on a 20 nm × 20 nm *x*–*y* grid from the mean square displacements over a timescale of 10 or 20 ms. Each point in the image is false‐colored according to the mean square displacement calculated over all displacements originating in a circle (*r* = 80 nm).

Co-movement analysis (eIF4E and eIF4G, ARC mRNA and eIF4E) was performed in MatLab R2014a (MathWorks) using Analyze2color (Analyze2color_diatrack3: https://github.com/timotheelionnet/Analyze2color)^24^. We localized particles and built trajectories in both channels separately. Trajectories that dwelled within 320 nm of one another for at least 10 ms were assigned as colocalized. To assess the colocalization statistics, we generated a matrix of the distances between all detected particles in both channels and computed the corresponding histogram^37^. We then normalized the distance histogram to account for the fact the area covered by each distance bin grows, and plotted the resulting normalized distribution, equivalent to the average density of channel 1 spots observed as a function of distance from channel 2 detections. If trajectories do not colocalize, we would randomly detect channel 1 spots at all positions in the cell without regard for channel 2 positions and therefore we would expect to observe a flat distribution. In the case of colocalization, we would expect an enrichment of short distances corresponding to comoving trajectories.

CDF curves were fit with a two-component fit (Eq. (1)) with *A* (fraction of molecules with the diffusion coefficient *D*_1_), 1*−A* (fraction of molecules with the diffusion coefficient *D*_2_), *D*_1_ (diffusion coefficient of the first diffusive species in μm^2^/s), *D*_2_ (diffusion coefficient of the second diffusive species in μm^2^/s), and *t* (frame time in s) as fitting parameters:

1 $$f\left(x\right)=1-\left[A*{{\rm {e}}}^{-{x}^{2}/4{D}_{1}t}+\left(1-A\right)*{{\rm {e}}}^{-{x}^{2}/4{D}_{2}t}\right]$$

### Fluorescence correlation spectroscopy (FCS)

FCS trajectories were obtained using a Leica TCS SP8 Falcon FLIM/FCS microscope with a Leica APO ×86/NA = 1.20 water-immersion objective equipped with a motorized correction collar (mottCorr, Leica). The microscope was controlled using Leica’s LAS X (3.5.7.23225) and LAS X FLIM/FCS (3.5.6) control software. Prior to the acquisition, cells at ~70–80% density were labeled with 100 nM JF585-Halo tag ligand^63^ and 200 nM JF646-SNAP-tag ligand^58^ for 10 and 45 min, respectively. Cells were incubated at 37 °C with 5% CO_2_ using a Tokai-hit stage top incubator. Just before each measurement, the mottCorr was adjusted using *xz*-scans in reflection mode to give the sharpest image of the living cell. *xz*-scans were also used to set the central plane of the cell. A set of two 10 s measurements were obtained, three in the nucleus of the cell, and three in the cytoplasm; between 10 and 20 cells were analyzed. The FCS and FCCS curves were exported and averaged using Prism 7 (GraphPad). Curve-fitting was conducted using IGOR Pro 9 (WaveMetrics). FCS curves were fitted to a one-component fit (Eq. (2) with the triplet state fraction of molecules (*T*), the lifetime of the triplet state (*Tt*, in ms), the structural parameter of the focal volume (*κ* = 5), and the diffusion time (*tau*, in ms) as fitting parameters:

2 $$G\left(\tau \right)=\left[1-T+T{e}^{-\tau /{Tt}}\right]{\left(1+\frac{\tau }{{tau}}\right)}^{-1}{\left(1+\frac{\tau }{{tau}*{\kappa }^{2}}\right)}^{-1/2}$$

FCS curves were also fitted to a two-component fit (Eq. (3)) with *T* (triplet state fraction of molecules), *Tt* (lifetime of the triplet state in ms), *A* (fraction of molecules with the diffusion time *tau*_1_), *κ* (structural parameter of the focal volume, *κ* = 5), *tau*_1_ (diffusion time of the first diffusive species in ms), and *tau*_2_ (diffusion time of the second diffusive species in ms) as parameters:

3 $$G\left(\tau \right)=	\left[1-T+T{{\rm {e}}}^{-\frac{\tau }{{Tt}}}\right]\left[{A*\left(1+\frac{\tau }{{{{\rm {tau}}}}_{1}}\right)}^{-1}{\left(1+\frac{\tau }{{{{\rm {tau}}}}_{1}*{\kappa }^{2}}\right)}^{-\frac{1}{2}}\right.\\ 	\left.+\,(1-A){*\left(1+\frac{{\rm {\tau}} }{{{{\rm {tau}}}}_{2}}\right)}^{-1}{\left(1+\frac{\tau }{{{{\rm {tau}}}}_{2}*{\kappa }^{2}}\right)}^{-1/2}\right]$$

### Cap pull-down assay, polysome profiling and ^35^S-Met/Cys labeling

Cap-pull down assay was performed as described in ref. 64. Briefly, cells at ~70% density were washed 3 times in ice-cold 1× PBS and scraped in Buffer A [50 mM Tris–HCl pH 7.5, 50 mM KCl, 1 mM EDTA, 0.5% sodium deoxycholate, 1 mM Vanadate, 1 mM sodium fluoride, 20 mM ß-Glycerophosphate]. Total proteins (500 μg) were incubated with 15 μL Immobilized γ-Aminophenyl-m7GTP (Jena Bioscience, #AC-155) at 4 °C for 3 h. Eluted material was analyzed by western blotting.

Polysome profiling was performed as described in ref. 65. 5–10 ODs of total RNA, measured at 254 nm, was loaded on 5–50% sucrose gradients. Sucrose gradients were displaced with 60% sucrose and the absorbance was recorded continuously at 254 nm using a Brandel BR‐188 density gradient fractionation system. ^35^S-Met/Cys labeling was performed as described in ref. 16.

### Reporting summary

Further information on research design is available in the Nature Research Reporting Summary linked to this article.

## Supplementary information

Supplementary Information

Description of Additional Supplementary Files

Supplementary Movie 1

Supplementary Movie 2

Supplementary Movie 3

Supplementary Movie 4

Supplementary Movie 5

Supplementary Movie 6

Supplementary Movie 7

Supplementary Movie 8

Reporting Summary

## Data Availability

The data that support this study are available from the corresponding authors upon reasonable request. Source Data are provided in this paper.

## Source data

Source data

## Code availability

We present a new Fiji plugin: Fiji plugin now allows for nearest-neighbor interpolation that does not change actual pixel intensities and thereby preserves the original noise distribution (as compared to linear or other higher-order interpolation schemes). This update is available via the Fiji Updater (http://fiji.sc/Downloads). It can be found under Plugins > Registration > Descriptor based Registration (2d/3d) and Plugins > Registration > Descriptor based Series Registration (2d/3d + t). The co-movement analysis code Analyze2color has now been deposited on GitHub:

(Analyze2color_diatrack3) https://github.com/timotheelionnet/Analyze2color.

## References

[1] Topisirovic I, Svitkin YV, Sonenberg N, Shatkin AJ (2011). Cap and cap-binding proteins in the control of gene expression. Wiley Interdiscip. Rev. RNA.

[2] Gingras AC (1999). Regulation of 4E-BP1 phosphorylation: a novel two-step mechanism. Genes Dev..

[3] Mader S, Lee H, Pause A, Sonenberg N (1995). The translation initiation factor eIF-4E binds to a common motif shared by the translation factor eIF-4 gamma and the translational repressors 4E-binding proteins. Mol. Cell. Biol..

[4] Kim SA, Heinze KG, Schwille P (2007). Fluorescence correlation spectroscopy in living cells. Nat. Methods.

[5] Bacia K, Kim SA, Schwille P (2006). Fluorescence cross-correlation spectroscopy in living cells. Nat. Methods.

[6] Grüner S (2016). The structures of eIF4E-eIF4G complexes reveal an extended interface to regulate translation initiation. Mol. Cell.

[7] Truitt ML (2015). Differential requirements for eIF4E dose in normal development and cancer. Cell.

[8] Shav-Tal Y (2004). Dynamics of single mRNPs in nuclei of living cells. Science.

[9] Averous J, Fonseca BD, Proud CG (2008). Regulation of cyclin D1 expression by mTORC1 signaling requires eukaryotic initiation factor 4E-binding protein 1. Oncogene.

[10] Marcotrigiano J, Gingras AC, Sonenberg N, Burley SK (1997). Cocrystal structure of the messenger RNA 5’ cap-binding protein (eIF4E) bound to 7-methyl-GDP. Cell.

[11] Matsuo H (1997). Structure of translation factor eIF4E bound to m7GDP and interaction with 4E-binding protein. Nat. Struct. Biol..

[12] Alain T (2012). eIF4E/4E-BP ratio predicts the efficacy of mTOR targeted therapies. Cancer Res..

[13] Tahmasebi S (2014). Multifaceted regulation of somatic cell reprogramming by mRNA translational control. Cell Stem Cell.

[14] Baudendistel N, Müller G, Waldeck W, Angel P, Langowski J (2005). Two-hybrid fluorescence cross-correlation spectroscopy detects protein-protein interactions in vivo. Chemphyschem.

[15] Gandin V (2016). mTORC1 and CK2 coordinate ternary and eIF4F complex assembly. Nat. Commun..

[16] Morita M (2013). mTORC1 controls mitochondrial activity and biogenesis through 4E-BP-dependent translational regulation. Cell Metab..

[17] Woodcock HV (2019). The mTORC1/4E-BP1 axis represents a critical signaling node during fibrogenesis. Nat. Commun..

[18] Tsukumo Y, Alain T, Fonseca BD, Nadon R, Sonenberg N (2016). Translation control during prolonged mTORC1 inhibition mediated by 4E-BP3. Nat. Commun..

[19] Dowling RJO (2010). mTORC1-mediated cell proliferation, but not cell growth, controlled by the 4E-BPs. Science.

[20] Pyronnet S (1999). Human eukaryotic translation initiation factor 4G (eIF4G) recruits mnk1 to phosphorylate eIF4E. EMBO J..

[21] Korneeva NL, Song A, Gram H, Edens MA, Rhoads RE (2016). Inhibition of mitogen-activated protein kinase (MAPK)-interacting kinase (MNK) preferentially affects translation of mRNAs containing both a 5’-terminal cap and hairpin. J. Biol. Chem..

[22] Ries J, Schwille P (2012). Fluorescence correlation spectroscopy. BioEssays.

[23] Hess ST, Huang S, Heikal AA, Webb WW (2002). Biological and chemical applications of fluorescence correlation spectroscopy: a review. Biochemistry.

[24] Katz, Z. B. et al. Mapping translation ‘hot-spots’ in live cells by tracking single molecules of mRNA and ribosomes. *Elife* **5**, 10415 (2016). 10.7554/eLife.10415 PMC4764586 26760529

[25] Villa N, Do A, Hershey JWB, Fraser CS (2013). Human Eukaryotic initiation factor 4G (eIF4G) protein binds to eIF3c, -d, and -e to promote mRNA recruitment to the Ribosome*. J. Biol. Chem..

[26] Wang DO, Martin KC, Zukin RS (2010). Spatially restricting gene expression by local translation at synapses. Trends Neurosci..

[27] Santini, E. et al. Reducing eIF4E–eIF4G interactions restores the balance between protein synthesis and actin dynamics in fragile X syndrome model mice. *Sci. Signal.* **10**, eaan0665 (2017). 10.1126/scisignal.aan0665 PMC5858943 29114037

[28] Napoli I (2008). The fragile X syndrome protein represses activity-dependent translation through CYFIP1, a new 4E-BP. Cell.

[29] Jung M-Y, Lorenz L, Richter JD (2006). Translational control by neuroguidin, a eukaryotic initiation factor 4E and CPEB binding protein. Mol. Cell. Biol..

[30] Bardy C (2015). Neuronal medium that supports basic synaptic functions and activity of human neurons in vitro. Proc. Natl Acad. Sci. USA.

[31] Guzowski JF (2000). Inhibition of activity-dependent arc protein expression in the rat hippocampus impairs the maintenance of long-term potentiation and the consolidation of long-term memory. J. Neurosci..

[32] Panja D (2009). Novel translational control in Arc-dependent long term potentiation consolidation in vivo. J. Biol. Chem..

[33] Das S, Moon HC, Singer RH, Park HY (2018). A transgenic mouse for imaging activity-dependent dynamics of endogenous Arc mRNA in live neurons. Sci. Adv..

[34] Tahmasebi S, Khoutorsky A, Mathews MB, Sonenberg N (2018). Translation deregulation in human disease. Nat. Rev. Mol. Cell Biol..

[35] Tanenbaum ME, Gilbert LA, Qi LS, Weissman JS, Vale RD (2014). A protein-tagging system for signal amplification in gene expression and fluorescence imaging. Cell.

[36] Rodriguez AJ, Shenoy SM, Singer RH, Condeelis J (2006). Visualization of mRNA translation in living cells. J. Cell Biol..

[37] Halstead JM (2015). An RNA biosensor for imaging the first round of translation from single cells to living animals. Science.

[38] Morisaki T, Stasevich TJ (2018). Quantifying single mRNA translation kinetics in living cells. Cold Spring Harb. Perspect. Biol..

[39] Wu B, Buxbaum AR, Katz ZB, Yoon YJ, Singer RH (2015). Quantifying protein–mRNA interactions in single live cells. Cell.

[40] Gandin V (2016). nanoCAGE reveals 5’ UTR features that define specific modes of translation of functionally related MTOR-sensitive mRNAs. Genome Res..

[41] Fingar DC (2004). mTOR controls cell cycle progression through its cell growth effectors S6K1 and 4E-BP1/Eukaryotic translation initiation factor 4E. Mol. Cell. Biol..

[42] Lahr, R. M. et al. La-related protein 1 (LARP1) binds the mRNA cap, blocking eIF4F assembly on TOP mRNAs. *Elife* **6**, 24146 (2017). 10.7554/eLife.24146 PMC5419741 28379136

[43] Cohen N (2001). PML RING suppresses oncogenic transformation by reducing the affinity of eIF4E for mRNA. EMBO J..

[44] Lejbkowicz F (1992). A fraction of the mRNA 5’ cap-binding protein, eukaryotic initiation factor 4E, localizes to the nucleus. Proc. Natl Acad. Sci. USA.

[45] Davis MR, Delaleau M, Borden KLB (2019). Nuclear eIF4E stimulates 3’-end cleavage of target RNAs. Cell Rep..

[46] Culjkovic B, Topisirovic I, Skrabanek L, Ruiz-Gutierrez M, Borden KLB (2006). eIF4E is a central node of an RNA regulon that governs cellular proliferation. J. Cell Biol..

[47] Das, S., Vera, M., Gandin, V., Singer, R. H. & Tutucci, E. Intracellular mRNA transport and localized translation. *Nat. Rev. Mol. Cell Biol.*10.1038/s41580-021-00356-8 (2021). 10.1038/s41580-021-00356-8 PMC9346928 33837370

[48] Sonneveld S, Verhagen BMP, Tanenbaum ME (2020). Heterogeneity in mRNA Translation. Trends Cell Biol..

[49] Genuth NR, Barna M (2018). Heterogeneity and specialized functions of translation machinery: from genes to organisms. Nat. Rev. Genet..

[50] Amorim IS, Lach G, Gkogkas CG (2018). The role of the eukaryotic translation initiation factor 4E (eIF4E) in neuropsychiatric disorders. Front. Genet..

[51] Dufourt J (2021). Imaging translation dynamics in live embryos reveals spatial heterogeneities. Science.

[52] Tsai A (2017). Nuclear microenvironments modulate transcription from low-affinity enhancers. Elife.

[53] Bhat M (2015). Targeting the translation machinery in cancer. Nat. Rev. Drug Discov..

[54] Stern-Ginossar, N., Thompson, S. R., Mathews, M. B. & Mohr, I. Translational control in virus-infected cells. *Cold Spring Harb. Perspect. Biol.* **11**, a033001 (2019). 10.1101/cshperspect.a033001 PMC6396331 29891561

[55] English BP, Singer RH (2015). A three-camera imaging microscope for high-speed single-molecule tracking and super-resolution imaging in living cells. Proc. SPIE Int. Soc. Opt. Eng..

[56] Edelstein, A., Amodaj, N., Hoover, K., Vale, R. & Stuurman, N. Computer control of microscopes using µManager. *Curr. Protoc. Mol. Biol.* Chapter **14**, Unit14.20 (2010). 10.1002/0471142727.mb1420s92 PMC3065365 20890901

[57] Grimm JB, Brown TA, English BP, Lionnet T, Lavis LD (2017). Synthesis of Janelia fluor HaloTag and SNAP-Tag ligands and their use in cellular imaging experiments. Methods Mol. Biol..

[58] Grimm JB (2015). A general method to improve fluorophores for live-cell and single-molecule microscopy. Nat. Methods.

[59] Zheng Q (2019). Rational design of fluorogenic and spontaneously blinking labels for super-resolution imaging. ACS Cent. Sci..

[60] Grimm JB (2016). Bright photoactivatable fluorophores for single-molecule imaging. Nat. Methods.

[61] Smith CS (2015). Nuclear accessibility of β-actin mRNA is measured by 3D single-molecule real-time tracking. J. Cell Biol..

[62] Thompson RE, Larson DR, Webb WW (2002). Precise nanometer localization analysis for individual fluorescent probes. Biophys. J..

[63] Grimm JB (2017). A general method to fine-tune fluorophores for live-cell and in vivo imaging. Nat. Methods.

[64] Gorrini C (2005). Fibronectin controls cap-dependent translation through β1 integrin and eukaryotic initiation factors 4 and 2 coordinated pathways. Proc. Natl Acad. Sci. USA.

[65] Gandin, V. et al. Polysome fractionation and analysis of mammalian translatomes on a genome-wide scale. *J. Vis. Exp.*10.3791/51455 (2014). 10.3791/51455 PMC4189431 24893926

