# Supplementary material for: Cap-dependent translation initiation monitored in living cells
Source: Nat Commun. 2022 Nov 2;13:6558. doi: 10.1038/s41467-022-34052-8 (PMC9630388; doi:10.1038/s41467-022-34052-8)
Supplement: Supplementary file 1 — Supplementary Information [file 41467_2022_34052_MOESM1_ESM.pdf]

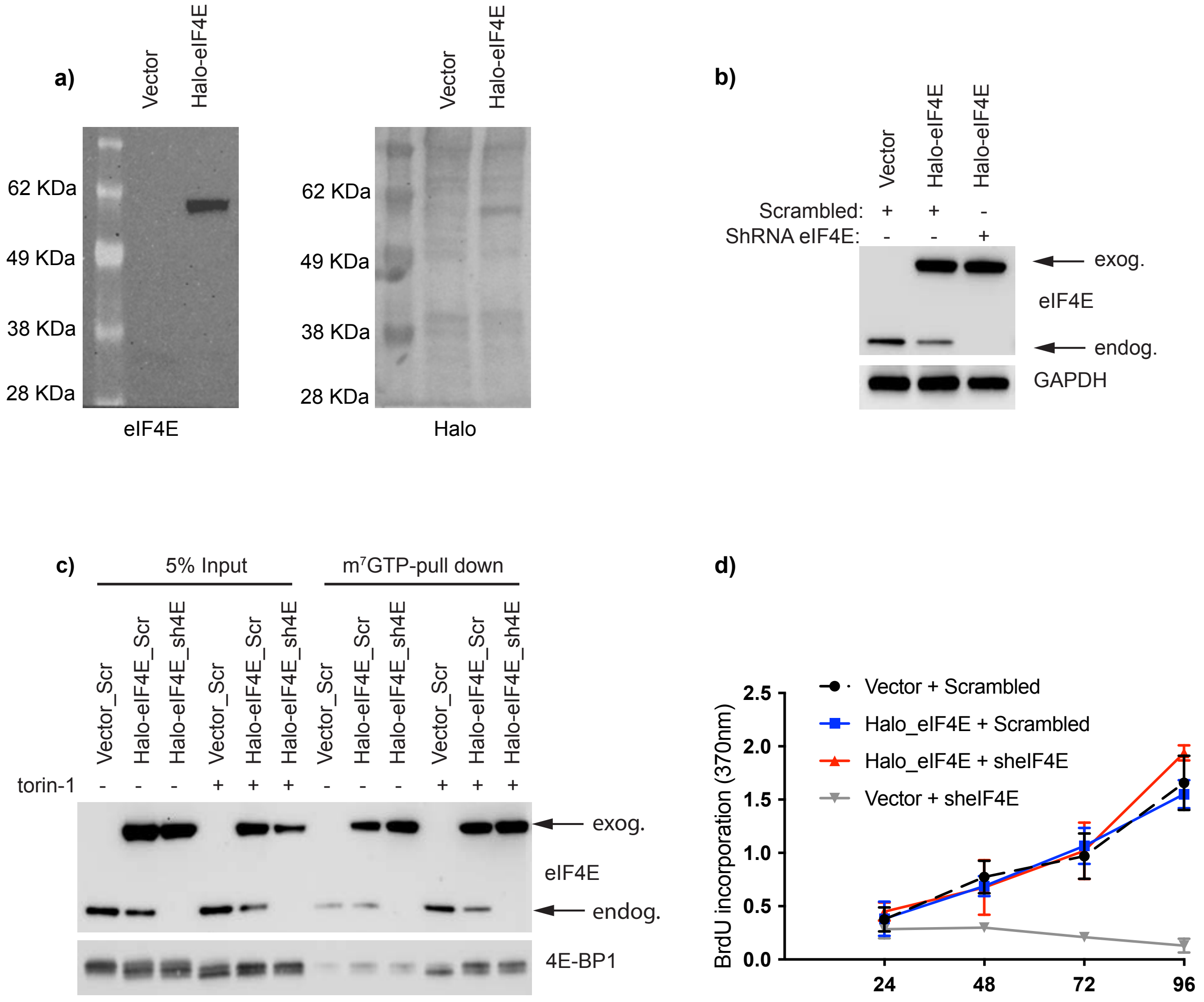
a)
Vector
Halo-eIF4E
62 KDa
49 KDa
38 KDa
28 KDa
eIF4E
Vector
Halo-eIF4E
62 KDa
49 KDa
38 KDa
28 KDa
Halo
b)
Vector
Halo-eIF4E
Halo-eIF4E
Scrambled: + + -
ShRNA eIF4E: - - +
exog.
eIF4E
endog.
GAPDH
c)
5% Input
m7GTP-pull down
Vector_Scr
Halo-eIF4E_Scr
Halo-eIF4E_sh4E
Vector_Scr
Halo-eIF4E_Scr
Halo-eIF4E_sh4E
Vector_Scr
Halo-eIF4E_Scr
Halo-eIF4E_sh4E
Vector_Scr
Halo-eIF4E_Scr
Halo-eIF4E_sh4E
torin-1 - - - + + + - - - + + +
exog.
eIF4E
endog.
4E-BP1
d)
Vector + Scrambled
Halo_eIF4E + Scrambled
Halo_eIF4E + sheIF4E
Vector + sheIF4E
BrdU incorporation (370nm)
2.5
2.0
1.5
1.0
0.5
0.0
24
48
72
96

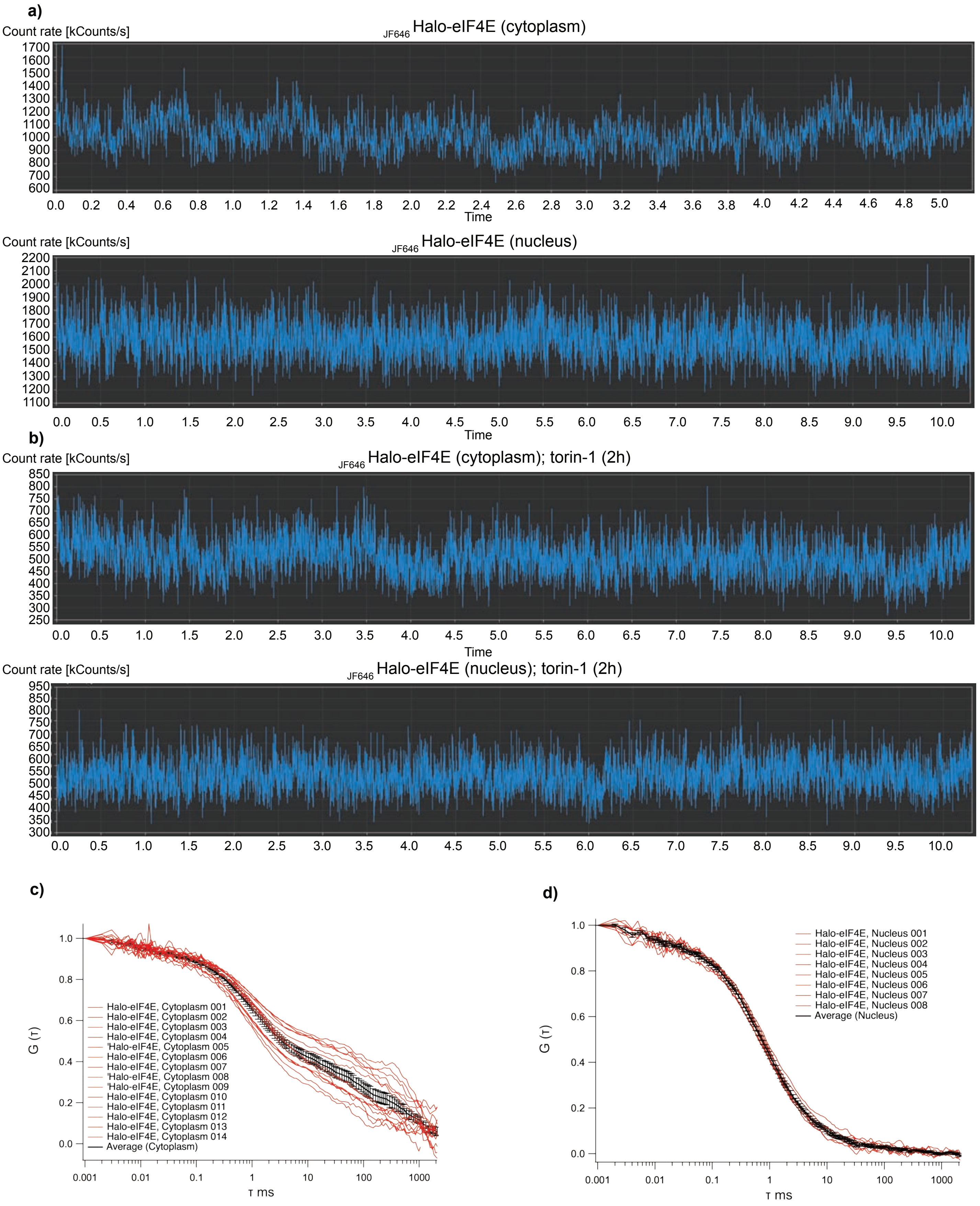

a)
Count rate [kCounts/s]
JF646 Halo-eIF4E (cytoplasm)
Time
Count rate [kCounts/s]
JF646 Halo-eIF4E (nucleus)
Time
b)
Count rate [kCounts/s]
JF646 Halo-eIF4E (cytoplasm); torin-1 (2h)
Time
Count rate [kCounts/s]
JF646 Halo-eIF4E (nucleus); torin-1 (2h)
c)
G (τ)
τ ms
Halo-eIF4E, Cytoplasm 001
Halo-eIF4E, Cytoplasm 002
Halo-eIF4E, Cytoplasm 003
Halo-eIF4E, Cytoplasm 004
'Halo-eIF4E, Cytoplasm 005
Halo-eIF4E, Cytoplasm 006
Halo-eIF4E, Cytoplasm 007
'Halo-eIF4E, Cytoplasm 008
'Halo-eIF4E, Cytoplasm 009
Halo-eIF4E, Cytoplasm 010
Halo-eIF4E, Cytoplasm 011
Halo-eIF4E, Cytoplasm 012
Halo-eIF4E, Cytoplasm 013
Halo-eIF4E, Cytoplasm 014
Average (Cytoplasm)
d)
G (τ)
τ ms
Halo-eIF4E, Nucleus 001
Halo-eIF4E, Nucleus 002
Halo-eIF4E, Nucleus 003
Halo-eIF4E, Nucleus 004
Halo-eIF4E, Nucleus 005
Halo-eIF4E, Nucleus 006
Halo-eIF4E, Nucleus 007
Halo-eIF4E, Nucleus 008
Average (Nucleus)

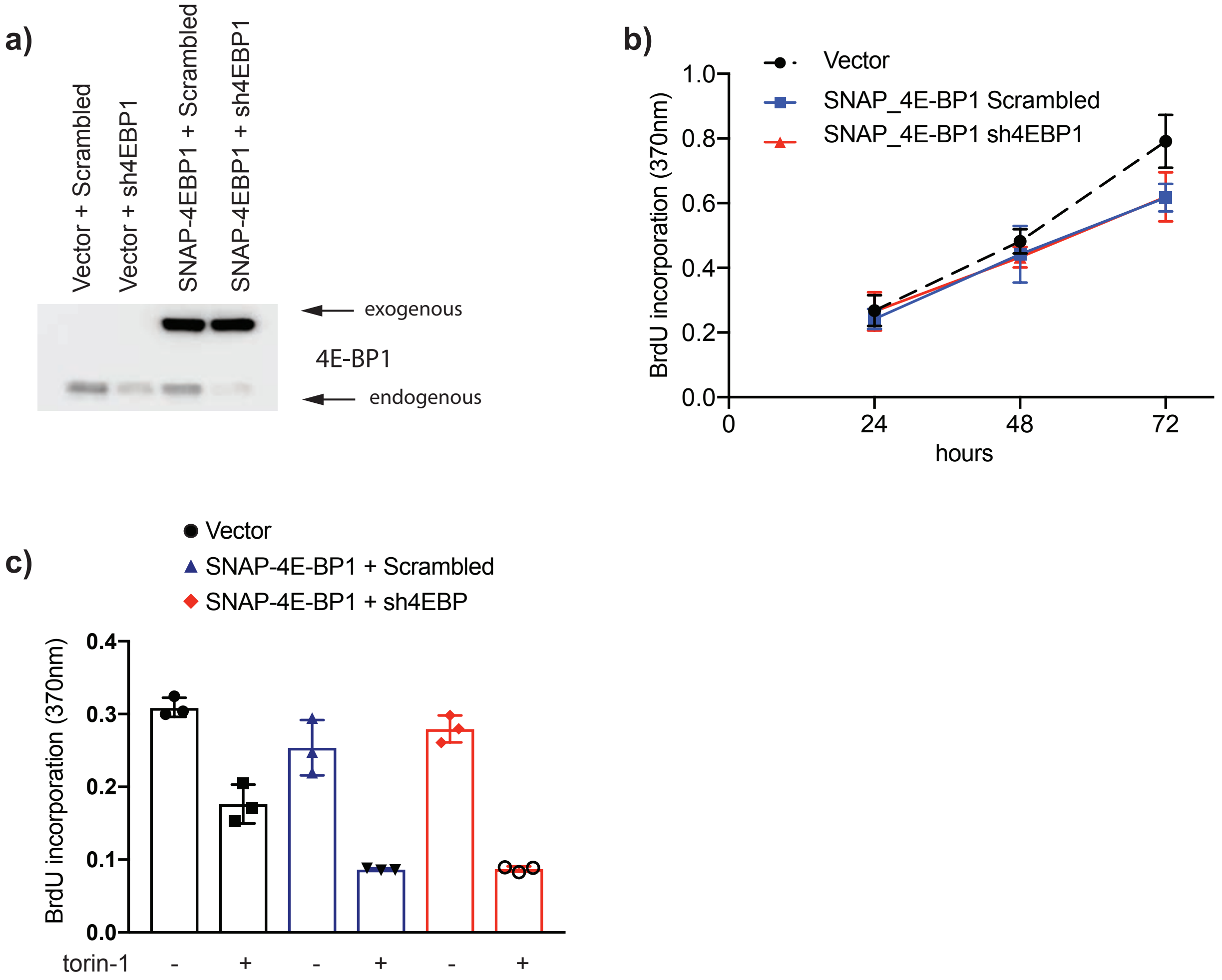
a)
Vector + Scrambled
Vector + sh4EBP1
SNAP-4EBP1 + Scrambled
SNAP-4EBP1 + sh4EBP1
exogenous
4E-BP1
endogenous
b)
Vector
SNAP_4E-BP1 Scrambled
SNAP_4E-BP1 sh4EBP1
BrdU incorporation (370nm)
hours
c)
Vector
SNAP-4E-BP1 + Scrambled
SNAP-4E-BP1 + sh4EBP
BrdU incorporation (370nm)
torin-1

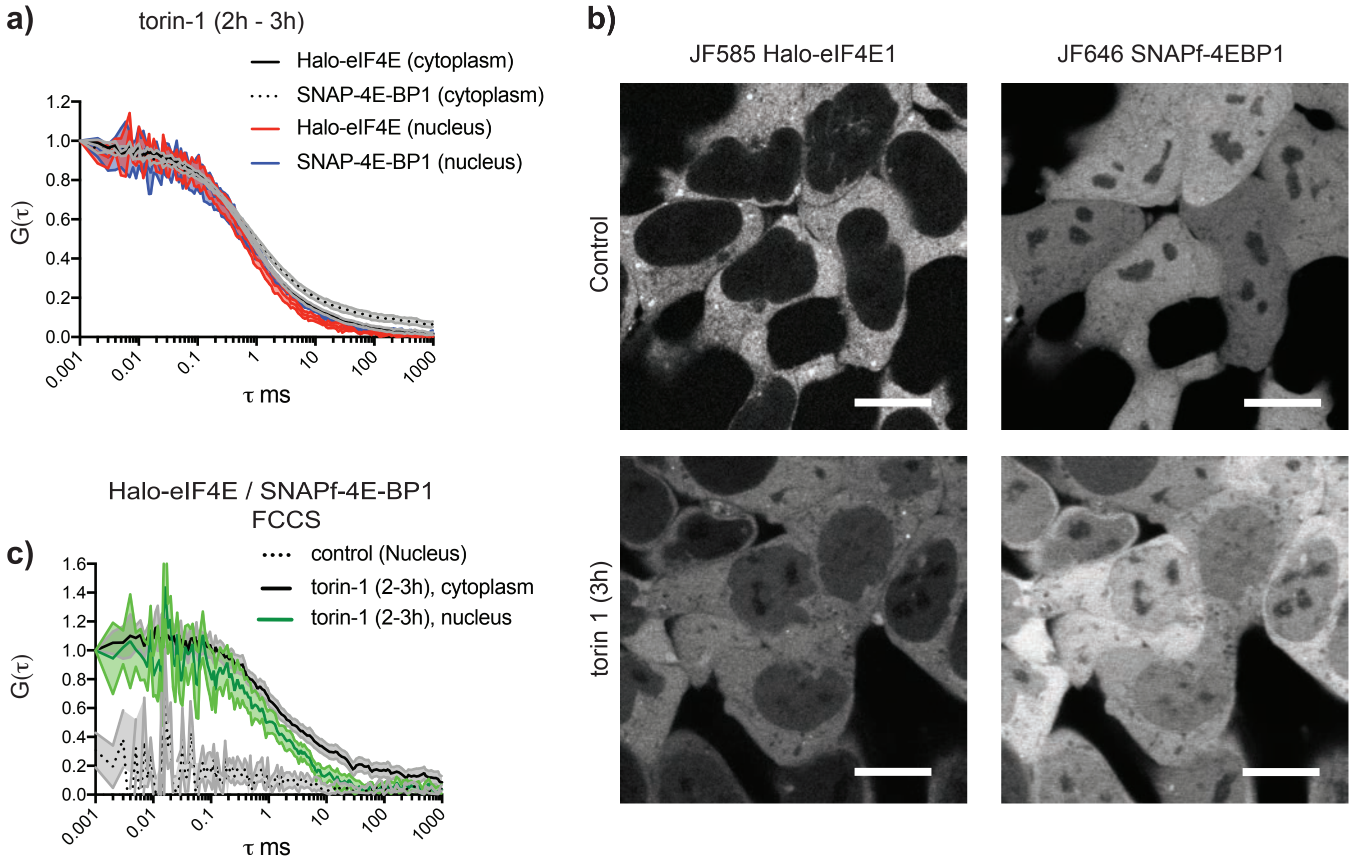
a)
torin-1 (2h - 3h)
Halo-eIF4E (cytoplasm)
SNAP-4E-BP1 (cytoplasm)
Halo-eIF4E (nucleus)
SNAP-4E-BP1 (nucleus)
G(τ)
τ ms
b)
JF585 Halo-eIF4E1
JF646 SNAPf-4EBP1
Control
torin 1 (3h)
Halo-eIF4E / SNAPf-4E-BP1
FCCS
c)
control (Nucleus)
torin-1 (2-3h), cytoplasm
torin-1 (2-3h), nucleus
G(τ)
τ ms

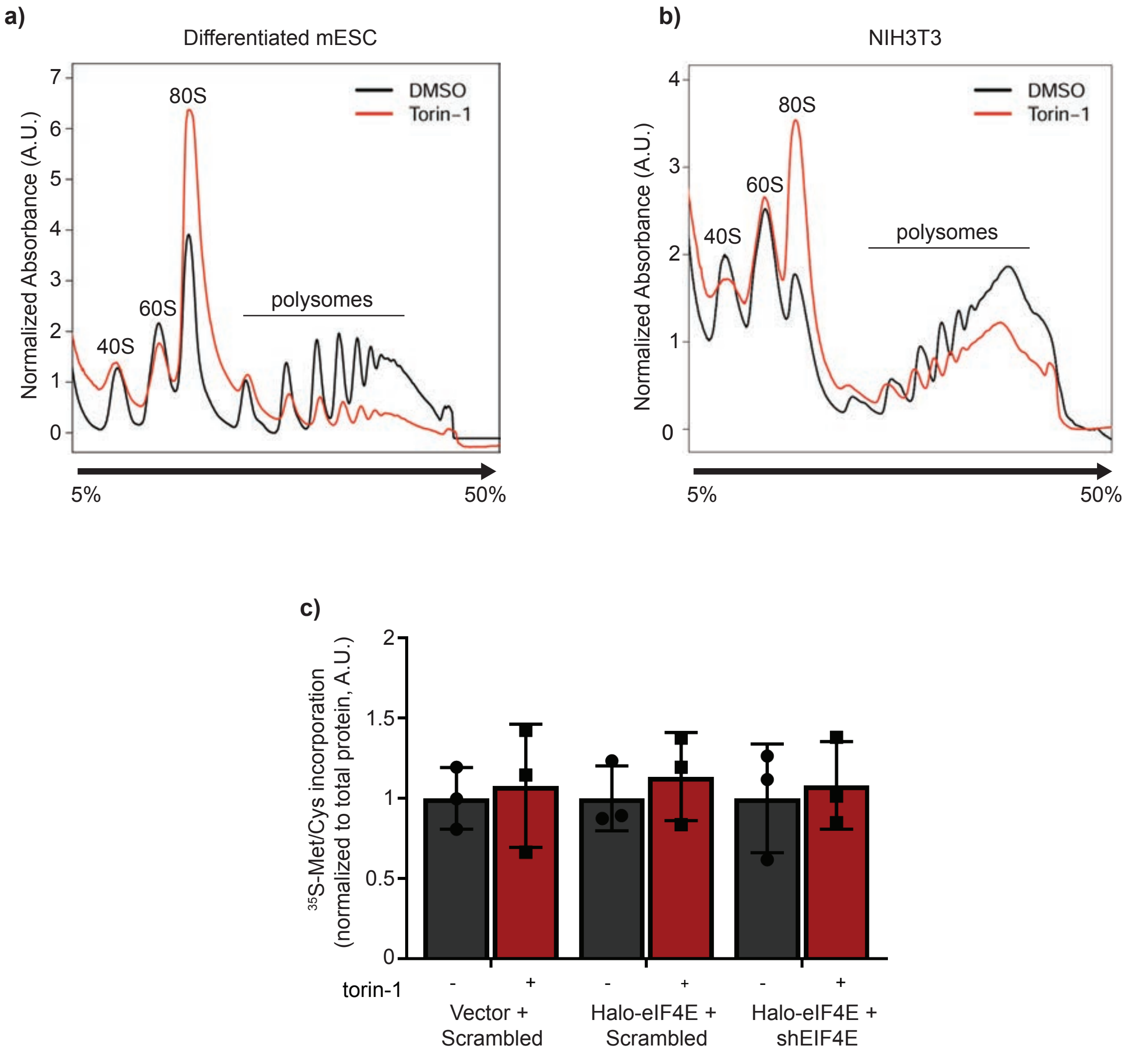
a)
Differentiated mESC
Normalized Absorbance (A.U.)
7
6
5
4
3
2
1
0
80S
60S
40S
polysomes
DMSO
Torin-1
5%
50%
b)
NIH3T3
Normalized Absorbance (A.U.)
4
3
2
1
0
80S
60S
40S
polysomes
DMSO
Torin-1
5%
50%
c)
35S-Met/Cys incorporation
(normalized to total protein, A.U.)
2
1.5
1
0.5
0
torin-1
-
+
-
+
-
+
Vector +
Scrambled
Halo-eIF4E +
Scrambled
Halo-eIF4E +
shEIF4E

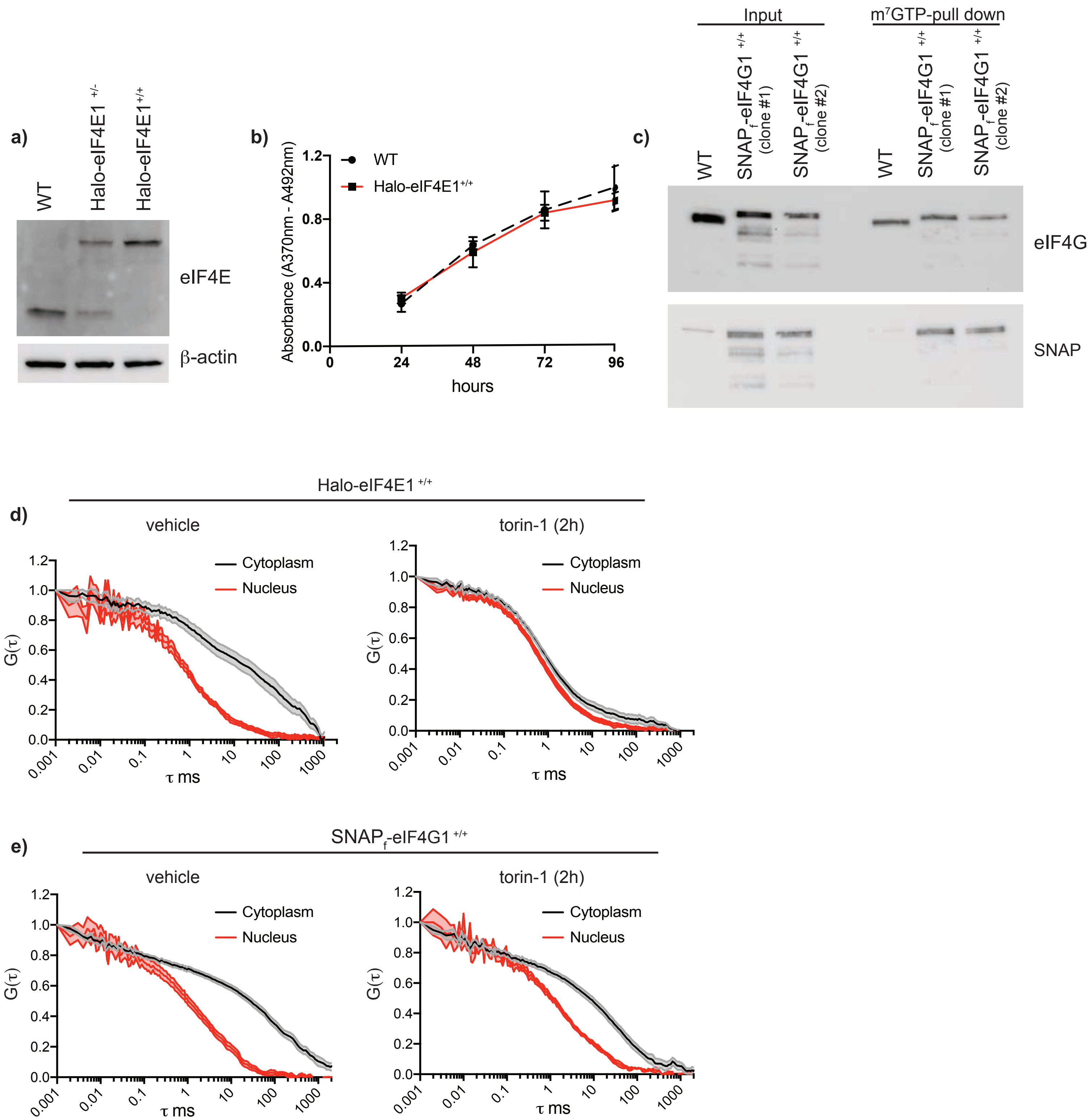
a)
WT
Halo-eIF4E1 +/-
Halo-eIF4E1+/+
eIF4E
β-actin
b)
WT
Halo-eIF4E1+/+
Absorbance (A370nm - A492nm)
1.2
0.8
0.4
0.0
0
24
48
72
96
hours
c)
Input
m7GTP-pull down
WT
SNAPf-eIF4G1 +/+ (clone #1)
SNAPf-eIF4G1 +/+ (clone #2)
eIF4G
SNAP
d)
Halo-eIF4E1 +/+
vehicle
torin-1 (2h)
Cytoplasm
Nucleus
G(τ)
τ ms
0.001
0.01
0.1
1
10
100
1000
e)
SNAPf-eIF4G1 +/+
vehicle
torin-1 (2h)
Cytoplasm
Nucleus
G(τ)
τ ms

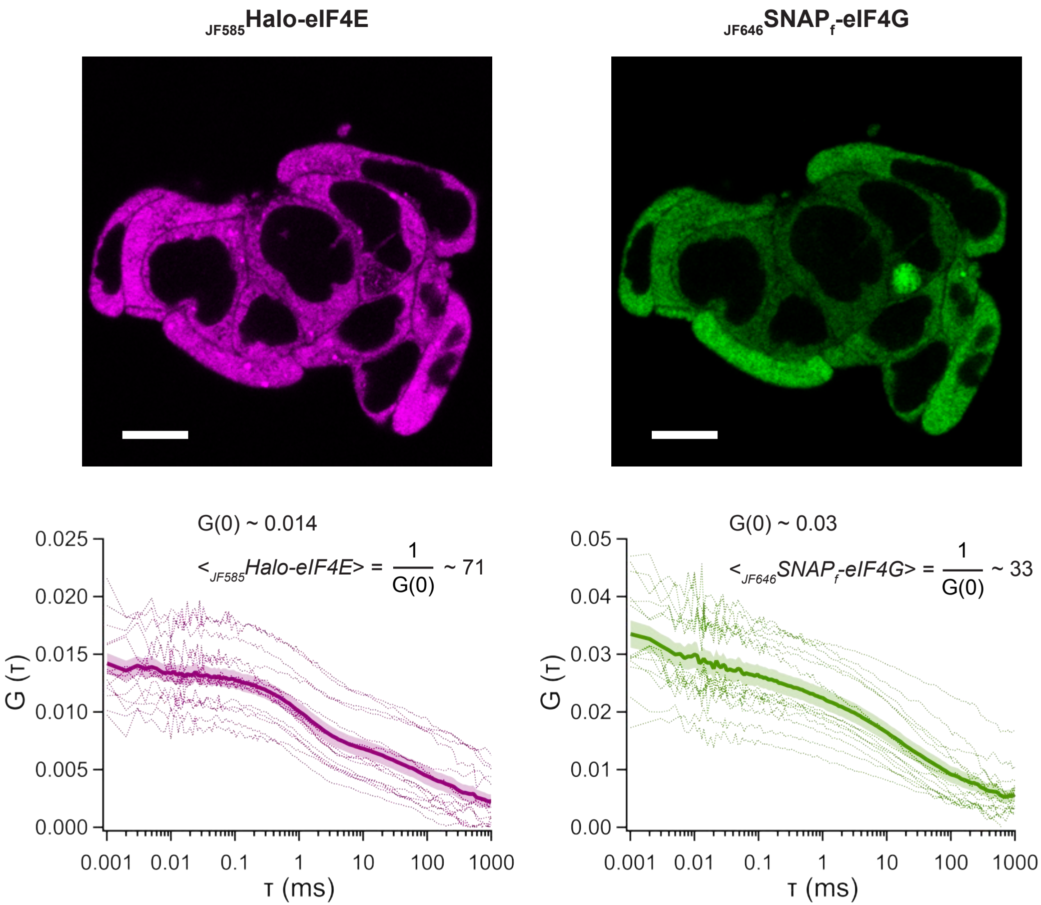

JF585Halo-eIF4E
JF646SNAPf-eIF4G
G(0) ~ 0.014
<JF585Halo-eIF4E> = 1/G(0) ~ 71
G (τ)
0.025
0.020
0.015
0.010
0.005
0.000
0.001 0.01 0.1 1 10 100 1000
τ (ms)
G(0) ~ 0.03
<JF646SNAPf-eIF4G> = 1/G(0) ~ 33
0.05
0.04
0.03
0.02
0.01
0.00

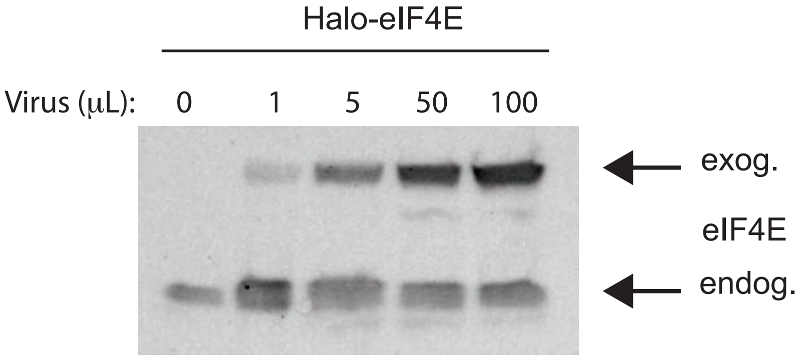
Halo-eIF4E
Virus (μL):
0
1
5
50
100
exog.
eIF4E
endog.

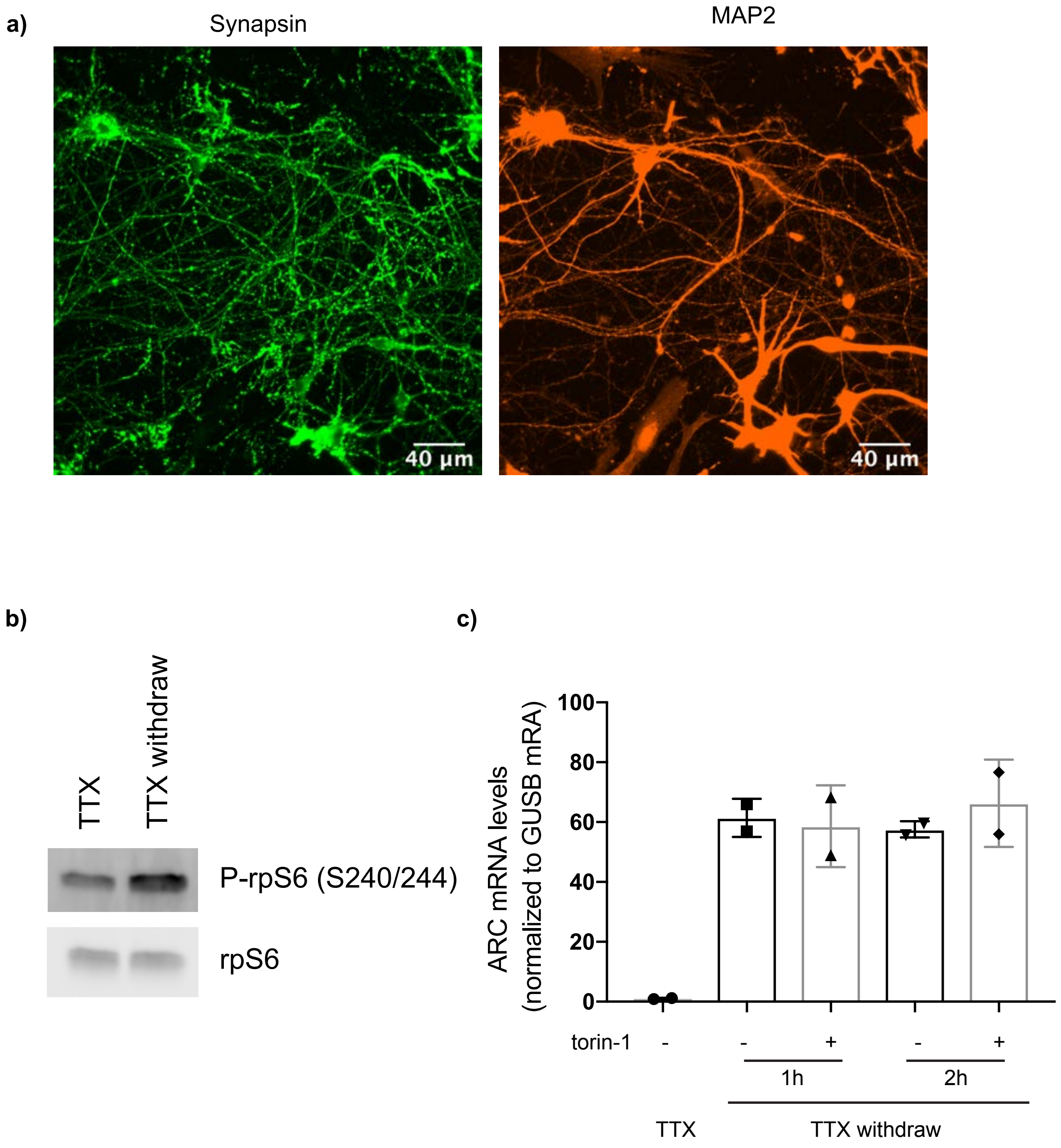
a)
Synapsin
MAP2
40 µm
40 µm
b)
TTX
TTX withdraw
P-rpS6 (S240/244)
rpS6
c)
ARC mRNA levels
(normalized to GUSB mRA)
100
80
60
40
20
0
torin-1
-
-
+
-
+
1h
2h
TTX
TTX withdraw

Statistical Analysis

for Fig 4g:

Student t-test: two mean values of two distributions:

alpha = 0.001:

Sample 1: eiF4E
Number of Points=355730
Mean=0.4932
Stdv=0.309552
Degrees of Freedom=355729

Sample 2: eIF4G
Number of Points=462072
Mean=0.337417
Stdv=0.231293
Degrees of Freedom=462071

Combined effective degrees of freedom: 636684
T-Test Statistic: 251.021
Lower Critical Value: -3.29054
Upper Critical Value: 3.29054
H0: avg1=avg2 H0 Acceptance range (0.0005,0.9995) **==> H0 Rejected**

C.I. mean1=[0.493197,0.493203] C.I. mean2=[0.337415,0.337419]

alpha = 0.001:

Sample 1: eIF4E
Number of Points=355730
Mean=0.4932
Stdv=0.309552
Degrees of Freedom=355729

Sample 2: comoving eIF4E/eiF4G
Number of Points=7947
Mean=0.318147
Stdv=0.601916
Degrees of Freedom=7946

Combined effective degrees of freedom: 8040.17
T-Test Statistic: 25.8498
Lower Critical Value: -3.29174
Upper Critical Value: 3.29174
H0: avg1=avg2 H0 Acceptance range (0.0005,0.9995) **==> H0 Rejected**

C.I. mean1=[0.493197,0.493203] C.I. mean2=[0.317897,0.318396]

alpha = 0.01:

Sample 1: eIF4G
Number of Points=462072
Mean=0.337417
Stdv=0.231293
Degrees of Freedom=462071

Sample 2: comoving eIF4E/eIF4G
Number of Points=7947
Mean=0.318147
Stdv=0.601916
Degrees of Freedom=7946

Combined effective degrees of freedom: 7986.41
T-Test Statistic: 2.85038
Lower Critical Value: -2.57645
Upper Critical Value: 2.57645
H0: avg1=avg2 H0 Acceptance range (0.005,0.995) **==> H0 Rejected**

C.I. mean1=[0.337416,0.337418] C.I. mean2=[0.317951,0.318342]

for Fig 6a:

Student t-test: two mean values of two distributions:

alpha = 0.001:

Sample 1: eIF4E
Number of Points=252266
Mean=0.781231
Stdv=0.742717
Degrees of Freedom=252265

Sample 2: eIF4G
Number of Points=318077
Mean=0.474816
Stdv=0.637885
Degrees of Freedom=318076

Combined effective degrees of freedom: 498459
T-Test Statistic: 164.589
Lower Critical Value: -3.29055
Upper Critical Value: 3.29055
H0: avg1=avg2 H0 Acceptance range (0.0005,0.9995) **==> H0 Rejected**

C.I. mean1=[0.781222,0.781241] C.I. mean2=[0.47481,0.474823]

Supplementary Figure Legends

Supplementary Fig. 1 Halo-eIF4E rescue cell proliferation and binds the 5'cap mRNA.

a) Vector control or Halo-eIF4E expressed in NIH3T3 cells. Total cell lysates were analyzed by western blotting with eIF4E and Halo-tag antibodies. No degradation products were detected. **b)** Vector control or Halo-eIF4E expressed in NIH3T3 cells infected with scrambled shRNA control (Scrambled) or shRNA targeting eIF4E (shRNA eIF4E). Total cell lysates were analyzed by western blotting. eIF4E antibodies detect both endogenous and exogenous eIF4E as indicated by the arrows. GAPDH was used as loading control. **c)** Cells described in a) were treated with DMSO (vehicle) or torin-1 for 1 hour. Total cell lysates (Input) were subjected to m7GTP-pull down assay and analyzed with the indicated antibodies. The Halo tag does not prevent eIF4E binding to the 5'cap analogue or 4E-BP1 binding upon mTOR inhibition. **d)** Proliferation of the indicated cells was measured by 5-bromo-2'-deoxyuridine (BrdU) incorporation. The results are represented as mean absorbance at 370 nm ± s.d. from three independent experiments

Supplementary Fig. 2 Slow diffusion of Halo-eIF4 in the cytoplasm of translating cells detected by FCS.

NIH3T3 cells that express Halo-eIF4E, in which the endogenous counterpart was knocked down by shRNA, were treated with DMSO (control) or 250nM torin-1 for 2 hours and subjected to FCS. **a,b)** Representative examples of $_{JF646}$Halo-eIF4E fluorescent fluctuations throughout the focal volume, in the cytoplasm and in the nucleus and in the indicated conditions. Major fluctuations (see within rectangle for representative example) are only detected in the cytoplasm of translating cells. **c,d)** Individual autocorrelations (in red) of the averaged autocorrelations (in black) depicting the temporal diffusion of eIF4E in the cytoplasm (left panel) and nucleus (right panel).

Supplementary Fig. 3 Overexpression SNAP$_f$-4E-BP1 slowed down cell proliferation and increased torin-1 efficacy.

a) Vector control or SNAPf-4E-BP1 were expressed in NIH3T3 cells infected with scrambled shRNA control (Scrambled) or shRNA targeting 4EBP1 (sh4EBP1). Total cell lysates were analyzed by western blotting. 4E-BP1 antibodies detect both endogenous and exogenous 4E-BP1 as indicated by the arrows. **b)** Proliferation of NIH3T3 cells infected with scrambled shRNA control (Scrambled) or shRNA targeting endogenous 4EBP1 (sh4EBP1) that express Vector control or SNAPf-4E-BP1 was measured by 5-bromo-2'-deoxyuridine (BrdU) incorporation. The results are represented as mean absorbance at 370 nm ± s.d. from three independent experiments. SNAPf-4E-BP1 overexpression slightly decreased cell proliferation at 48 and 72 hours. **c)** Cells described in a) were treated with vehicle control (DMSO) or 250nM torin-1 for 16 hours. Proliferation was measured as in b). The results are represented as mean absorbance at 370 nm ± s.d. from three independent experiments. 4E-BP1 overexpression increase the cytostatic effect of torin-1.

Supplementary Fig. 4 eIF4E:4E-BP1 complexes accumulate in the nucleus upon prolonged mTOR inhibition.

a-c) Differentiated mESC in which Halo and SNAPf tag were inserted into the Eif4e and Eif4ebp1 locus, respectively, were treated with vehicle (DMSO) or 250nM torin-1 from 2 to 3 hours. Halo-eIF4E and Snapf-4E-BP1 autocorrelations diffuse as fast as the nuclear counterparts in the indicated conditions (a). Data are presented as a mean value +/- SEM. Representative field of view of differentiated mESC showing subcellular distribution of $_{JF585}$Halo-eIF4E and $_{JF646}$Snapf-4E-BP1 in control and torin-1 treated cells. Images depicted distribution of initiation factors in living cells (b). eIF4E translocate to the nucleus 3 hours upon mTOR inhibition. Simultaneous diffusion of $_{JF585}$Halo-eIF4E and $_{JF646}$Snapf-4E-BP1 analyzed in differentiated mESC by fluorescent cross-correlation spectroscopy (FCCS) in the indicated conditions.
Cross-correlation was detected in the cytoplasm and in the nucleus 3 hours upon mTOR inhibition. No cross-correlation was observed in the nucleus of translating cells (control). Data are presented as mean values +/- SEM (c).

Supplementary Fig. 5 Impaired translation initiation due to eIF4E release from the 5'cap with no major difference in global translation.
a,b) Differentiated mESC Halo-eIF4E$^{+/+}$ (a) and NIH3T3 (b) that express exogenous Halo-eIF4E were treated with DMSO (control) or 250nM torin-1 for 2 hours. Cytosolic extracts were sedimented by centrifugation on 5-50% sucrose gradients. Free ribosomal subunits (40S and 60S), monosomes (80S) and translating ribosomes (polysome) are indicated. Increased in the 80S pick and decreased polysome levels showed initiation defects upon mTOR inhibition. Global protein synthesis measure by 35S-Met/Cys incorporation in the indicated cell lines treated with DMSO (-) or torin-1 (+) for 2 hours **(c)**. 35S-Met/Cys incorporation was normalized by total proteins and expressed as arbitrary units (A.U). Each replicate is represented as a single point on the corresponding bar graph (n=3). Data are presented as a mean values +/- SD). Expression of Halo-eIF4E does not sensitize the cells to torin-1 treatment.

Supplementary Fig. 6 Tagging of endogenous translation factors does not affect mESC viability or their binding dynamics.
a) Halo-tag inserted in the endogenous locus of EIF4E by Crispr/cas9. Total cell lysates from parental, heterozygote and homozygote cells were analyzed by western blotting using eIF4E antibodies. β-actin was used as a loading control. Heterozygote (Halo-eIF4E+/- or homozygote insertion (Halo-eIF4E$^{+/+}$) is shown. **b)** Proliferation of parental and Halo-eIF4E homozygote (Halo-eIF4E$^{+/+}$) differentiated to fibroblasts was measured by 5-bromo-2′-deoxyuridine (BrdU) incorporation. The results are represented as mean absorbance at 370 nm ± s.d. from three independent experiments. **c)** SNAP$_f$-tag was inserted in the endogenous locus of EIF4G1. Total cell lysates (input) from parental and eIF4G1 homozygote differentiated mESC (eIF4G1$^{+/+}$) were subjected to m7GTP-pull down assay and analyzed by western blotting using the indicated antibodies. **d,e)** Autocorrelation of endogenous $_{JF646}$Halo-eIF4E1 and $_{JF646}$SNAPf-eIF4G1 in cells described above. Data are presented as mean values +/- SEM). Cells were treated with DMSO (control) or 250nM torin-1 for 2 hours (2h). eIF4E and eIF4G1 molecules diffuse slower in the cytoplasmic of translating cells as compared to nuclear counterpart. Upon torin-1 treatment (torin-1 (2h)), cytoplasmic eIF4E molecules, but not eIF4G, diffuse as fast as the nuclear counterpart.

Supplementary Fig. 7 Measured apparent eIF4E and eIF4G concentrations in double knock-in Halo-eIF4E$^{+/+}$/SNAPf-eIF4G$^{+/+}$ cells labeled with $_{JF585}$Halo-eIF4E and $_{JF646}$SNAPf-eIF4G.
(top) Representative field of view of double knock-in Halo-eIF4E$^{+/+}$/SNAP-eIF4G$^{+/+}$ mESCs showing subcellular distribution of $_{JF585}$Halo-eIF4E (in magenta) and $_{JF646}$Snapf-eIF4G (in green). Images depicted distribution of initiation factors in living cells (scale bar: 10 μm). (bottom) Simultaneous diffusion of $_{JF585}$Halo-eIF4E and $_{JF646}$Snapf-eIF4G analyzed by FCS. Non-normalized autocorrelation curves representing individual diffusion of $_{JF585}$Halo-eIF4E (in magenta) and $_{JF646}$SNAPf-eIF4G (in green) in the indicated conditions (N=14±S.E.M.). The average number of molecules <N> in a focal volume was calculated from the G(0) timepoints of the non-normalized FCS curves. <N> was determined to be 71 for $_{JF585}$Halo-eIF4E and 33 for $_{JF646}$Snapf-eIF4G. The calculated apparent concentrations are 249 nM for $_{JF585}$Halo-eIF4E and 113 nM for $_{JF646}$Snapf-eIF4G. Values of <N> in the range between 0.1 and 100 are well suited for FCS (Ries et al. 2012; Hess et al. 2022).

Supplementary Fig. 8 Expression of Halo-eIF4E in primary cortical neurons.
Primary cortical neurons were infected with 1μL, 5 μL, 50 μL and 100 μL of lentivirus carrying Halo-eIF4E (concentration at 2E^7 IU/mL). Protein extracts were analyzed by western blotting using anti-eIF4E antibodies. Endogenous and exogenous eIF4E are indicated. Single-particle tracking was performed in neurons that express Halo-eIF4E at a similar level as the endogenous counterpart (5 μL) to minimize overexpression artefacts in the neuronal processes.

Supplementary Fig. 9 mTOR inhibition does not affect ARC mRNA stability.
a) Differentiated neurons derived from mESC. Immunostaining with the pre-synaptic marker Synapsin and the neuronal marker MAP2 is shown. **b)** Cells described in a) were treated for 16 hours with TTX. After 16 hours, TTX was removed for 2 hours and protein extracts were analyzed by western blot with the indicated antibodies. TTX withdraw increased mTOR activity.
c) Neurons were activated by TTX withdrawal with and without 250nM torin-1 for 1 and 2 hours. Expression levels of the mature ARC mRNA, in the indicated conditions, were determined by RT-qPCR (n=3). Data are presented as mean values +/- SD). Values were normalized to the levels of the house-keeping GUSB mRNA.
