# Supplementary material for: Cap-dependent translation initiation monitored in living cells
Source: Nat Commun. 2022 Nov 2;13:6558. doi: 10.1038/s41467-022-34052-8 (PMC9630388; doi:10.1038/s41467-022-34052-8)
Supplement: Supplementary file 2 — Description of Additional Supplementary Files [file 41467_2022_34052_MOESM2_ESM.pdf]

<u>Description of Additional Supplementary Files</u>

Supplementary Movie 1- Single molecule Halo-eIF4E and SNAPf-eIF4G in NIH3T3 cells Simultaneous detection of JF646HaloeIF4E (magenta) and JF549SnapfeIF4G (green) single molecules in the cytoplasm of living NIH3T3 cells. Interframe intervals indicated. Scale bar = 8 µm.

Supplementary Movie 2- Single molecule Halo-eIF4E in dendritic branches of inactivated neurons Detection of JF646Halo-eIF4E single molecules in the dendrites of dissociated neurons treated with 1.5 µM TTX for 16h prior to imaging. Inter-frame intervals indicated.

Supplementary Movie 3- Single molecule Halo-eIF4E in dendritic branches of activated neurons Detection of JF646Halo-eIF4E single molecules in the dendrites of dissociated neurons activated with TTX withdrawal. Inter-frame intervals indicated.

Supplementary Movie 4. Single molecule Halo-eIF4E in dendritic branches of activated neurons upon mTOR inhibition. Detection of JF646Halo-eIF4E single molecules in the dendrites of neurons activated with TTX withdrawal in presence of 250nM torin-1. Interframe intervals indicated.

Supplementary Movie 5. Real-time tracking of Halo-eIF4E diffusion in a mature dendrite along dendritic spines. Detection of PA(JF646)HaloeIF4E single molecules in dendrite of dissociated neuron. Box highlight spine-enriched region. Inter-frame intervals indicated.

Supplementary Movie 6. Differential diffusion of Halo-eIF4E and SNAPfeIF4G in a dendrite of activated neurons. Simultaneous detection of JF549Halo-eIF4E (green) and JF646Snapf-eIF4G (magenta) single molecules in dendrites of mESCdifferentiated neurons. Inter-frame intervals indicated.

Supplementary Movie 7. Singlemolecule ARC mRNA and eIF4E in a mature dendrite. Simultaneous detection of JF646Halo-eIF4E (green) and ARC-PP7-mRNA labelled with PCP-GFP of differentiated/dissociated neuron. Inter-frame intervals indicated.

Supplementary Movie 8. Singlemolecule ARC mRNAs and eIF4E. Co-tracking of ARC mRNA/Halo-eIF4E molecules. Note the near static motion of the translation factor interacting with ARC-mRNA. Inter-frame intervals indicated. Scale bar: 1.6 µm.
