# Supplementary material for: Cap-dependent translation initiation monitored in living cells
Source: Nat Commun. 2022 Nov 2;13:6558. doi: 10.1038/s41467-022-34052-8 (PMC9630388; doi:10.1038/s41467-022-34052-8)
Supplement: Supplementary file 11 — Reporting Summary [file 41467_2022_34052_MOESM11_ESM.pdf]

nature portfolio

Corresponding author(s): NCOMMS-22-08684-T
Valentina Gandin
Robert H. Singer

Last updated by author(s): 2022/08/17

Reporting Summary

Nature Portfolio wishes to improve the reproducibility of the work that we publish. This form provides structure for consistency and transparency in reporting. For further information on Nature Portfolio policies, see our Editorial Policies and the Editorial Policy Checklist.

Statistics

For all statistical analyses, confirm that the following items are present in the figure legend, table legend, main text, or Methods section.

n/a	Confirmed	
☐	☒	The exact sample size (*n*) for each experimental group/condition, given as a discrete number and unit of measurement
☐	☒	A statement on whether measurements were taken from distinct samples or whether the same sample was measured repeatedly
☐	☒	The statistical test(s) used AND whether they are one- or two-sided<br>*Only common tests should be described solely by name; describe more complex techniques in the Methods section.*
☒	☐	A description of all covariates tested
☒	☐	A description of any assumptions or corrections, such as tests of normality and adjustment for multiple comparisons
☐	☒	A full description of the statistical parameters including central tendency (e.g. means) or other basic estimates (e.g. regression coefficient) AND variation (e.g. standard deviation) or associated estimates of uncertainty (e.g. confidence intervals)
☐	☒	For null hypothesis testing, the test statistic (e.g. *F*, *t*, *r*) with confidence intervals, effect sizes, degrees of freedom and *P* value noted<br>*Give P values as exact values whenever suitable.*
☒	☐	For Bayesian analysis, information on the choice of priors and Markov chain Monte Carlo settings
☒	☐	For hierarchical and complex designs, identification of the appropriate level for tests and full reporting of outcomes
☒	☐	Estimates of effect sizes (e.g. Cohen's *d*, Pearson's *r*), indicating how they were calculated

Our web collection on statistics for biologists contains articles on many of the points above.

Software and code

Policy information about availability of computer code

Data collection	for single particle tracking:<br>open-source software: microManager (1.4.20)<br>commercial software: LABVIEW 2012 (National Instruments)<br>for FCS measurements:<br>commercial software: Leica LAS X (3.5.7.23225) and Leica LAS X FLIM/FCS (3.5.6) control software
Data analysis	new Fiji plugin: Fiji plugin now allows for nearest-neighbor interpolation that does not change actual pixel intensities and thereby preserves the original noise distribution (as compared to linear or other higher-order interpolation schemes). This update is available via the Fiji Updater (http://fiji.sc/Downloads). It can be found under Plugins > Registration > Descriptor based Registration (2d/3d) and Plugins > Registration > Descriptor based Series Registration (2d/3d+t).<br>commercial software: IGOR Pro 8.04 and IGOR Pro 9 (WaveMetrics), MatLab R2014a (MAthworks), Prism 7 (Graphpad), DiaTrack 3.04 Pro<br>open-source software used: Fiji/ ImageJ2 (v.2.3.0/153q)<br>Analyze2color (Analyze2color_diatrack3: https://github.com/timotheelionnet/Analyze2color)

For manuscripts utilizing custom algorithms or software that are central to the research but not yet described in published literature, software must be made available to editors and reviewers. We strongly encourage code deposition in a community repository (e.g. GitHub). See the Nature Portfolio guidelines for submitting code & software for further information.

Data

Policy information about availability of data

All manuscripts must include a data availability statement. This statement should provide the following information, where applicable:

- Accession codes, unique identifiers, or web links for publicly available datasets
- A description of any restrictions on data availability
- For clinical datasets or third party data, please ensure that the statement adheres to our policy

Source data are provided with this paper.

Field-specific reporting

Please select the one below that is the best fit for your research. If you are not sure, read the appropriate sections before making your selection.

[x] Life sciences [ ] Behavioural & social sciences [ ] Ecological, evolutionary & environmental sciences

For a reference copy of the document with all sections, see nature.com/documents/nr-reporting-summary-flat.pdf

Life sciences study design

All studies must disclose on these points even when the disclosure is negative.

Sample size	For all live-cell FCS experiments after drug treatment, we collected as many measurements of different cells as was possible for any given time point. Measurements were also conducted on multiple days. We found that the molecular diffusion across several cells was highly reproducible, and we determined that we had collected more than enough data-points to give repetitive FCS curves with small standard errors.
Data exclusions	No data were excluded from the analysis.
Replication	All attempts of replication were successful and showed comparable results. The number of replicates in each experiment is detailed either in the figure or the figure legends.
Randomization	For cell culture experiments no randomization was necessary. Randomization is not relevant to our study. Pharmacological treatments were added to the cells right after data acquisition of the untreated sampleswithout removing the plates from the microscope to minimize sample variation and drifting.
Blinding	Blinding was not relevant to this study. Pharmacological treatments were added to the cells right after data acquisition of the untreated samples without removing the plates from the microscope to minimize sample variation and drifting.

Reporting for specific materials, systems and methods

We require information from authors about some types of materials, experimental systems and methods used in many studies. Here, indicate whether each material, system or method listed is relevant to your study. If you are not sure if a list item applies to your research, read the appropriate section before selecting a response.

Materials & experimental systems

n/a	Involved in the study	
[ ]	[x]	Antibodies
[ ]	[x]	Eukaryotic cell lines
[x]	[ ]	Palaeontology and archaeology
[ ]	[x]	Animals and other organisms
[x]	[ ]	Human research participants
[x]	[ ]	Clinical data
[x]	[ ]	Dual use research of concern

Methods

n/a	Involved in the study	
[x]	[ ]	ChIP-seq
[x]	[ ]	Flow cytometry
[x]	[ ]	MRI-based neuroimaging

Antibodies

Antibodies used	anti-eIF4E (Monoclonal (87/eIF-4E), BD Transduction Laboratories™ #610269), anti-4E-BP1 (#9644), anti-eIF4G1 (#2498), anti-phosho-4E-BP1 Thr37/46 (#2855), anti-phospho- 4E-BP1 Ser65 (#9451), antiphosho-rpS6 Ser 240/244 (#2215), anti-rpS6 (SantaCruz Biotechnology, #sc-74459) all from Cell Signaling Technology, anti-GAPDH (#G8795) and anti-b actin (#A5441) from Sigma-Aldrich, anti-ARC (SantaCruz Biotechnology, #sc-17839, anti-HaloTag (Promega, #G9211), anti-SNAPtag (New England Biolabs, #P9310S) and anti-Cyclin D1 (BD Bioscience, #556470). Secondary

	antibodies, rabbit (Sigma-Aldrich, GENA934) and mouse (Amersham,NA934) IgG HRP linked, were used at 1:5,000.
Validation	eIF4E <validated in https://www.bdbiosciences.com/en-us/products/reagents/microscopy-imaging-reagents/immunofluorescence-reagents/purified-mouse-anti-eif-4e.610270>. All antibodies from Cell Signaling Technology were validated by the manufacture and extensively used in the literature. anti-rpS6 from Santa Cruz was validated by the manufacture <https://www.scbt.com/p/ribosomal-protein-s6-antibody-c-8> and extensively used in the literature as described in their website (see provided link). Anti-GAPDH and b-actin Ab from Sigma were validated by manufacturers and used in the literature <https://www.sigmaaldrich.com/US/en/product/sigma/g8795>, <https://www.sigmaaldrich.com/US/en/product/sigma/a5441>. anti-Arc was validated by manufacturer and previously used in the literature see <https://www.scbt.com/p/arc-antibody-c-7>. Same for all the other antibodies used: Halo-tag <https://www.promega.com/-/media/files/resources/protocols/technical-manuals/0/halotag-technology-focus-on-imaging-protocol.pdf?rev=6943a74aefee4ea38937403e6e3bcdb9&sc_lang=en>; anti-SNAP <https://www.neb.com/products/p9310-anti-snap-tag-antibody-polyclonal#Product%20Information_Product%20Notes>; anti-Cyclin D1 <https://www.bdbiosciences.com/en-us/products/reagents/microscopy-imaging-reagents/immunohistochemistry-reagents/purified-mouse-anti-human-cyclin-d1.556470>.

Eukaryotic cell lines

Policy information about cell lines

Cell line source(s)	NIH3T3 were obtained from America Type Culture Collection (ATCC). JM8.N4 mouse ESCs from the C57BL/6N were from Liu laboratory, Janelia Research Campus.
Authentication	Cells were previously authenticated
Mycoplasma contamination	All cell lines used tested negative for mycoplasma
Commonly misidentified lines (See ICLAC register)	no commonly misidentified cells were used in this study

Animals and other organisms

Policy information about studies involving animals; ARRIVE guidelines recommended for reporting animal research

Laboratory animals	Mus musculus, STRAIN, females, 3 months old
Wild animals	*Provide details on animals observed in or captured in the field; report species, sex and age where possible. Describe how animals were caught and transported and what happened to captive animals after the study (if killed, explain why and describe method; if released, say where and when) OR state that the study did not involve wild animals.*
Field-collected samples	*For laboratory work with field-collected samples, describe all relevant parameters such as housing, maintenance, temperature, photoperiod and end-of-experiment protocol OR state that the study did not involve samples collected from the field.*
Ethics oversight	Primary rodent work was performed in accordance with protocols approved by the Janelia Research Campus Institutional Animal Care and Use Committee (IACUC) guidelines

Note that full information on the approval of the study protocol must also be provided in the manuscript.
